# Early mobilization in neurocritical care as a threshold-regulated intervention: a narrative review of mechanistic integration and implications for neurological recovery and physiological stability

**DOI:** 10.3389/fneur.2026.1837085

**Published:** 2026-09-29

**Authors:** Ying Hong, WenYan Hu, JiZu Tang, Feng Xiong

**Affiliations:** 1 Department of Neurology, Zhuzhou Central Hospital, Zhuzhou, Hunan, China; 2 Department of Orthopedics, Zhuzhou Central Hospital, Zhuzhou, Hunan, China

**Keywords:** early ambulation, critical care, nervous system diseases, cerebral blood flow, intracranial pressure, neuromuscular diseases, hemodynamics, physiologic

## Abstract

The integration of early mobilization into neurocritical care remains constrained by uncertainty regarding how heterogeneous physiological systems respond to graded activity during neurological instability. Although early mobilization is increasingly used in intensive care, the relationships between intervention timing, intensity, neurological recovery, functional outcomes, and physiological tolerance remain incompletely defined in acute neurocritical populations. This narrative review integrates clinical and mechanistic evidence to examine how immobility and reactivation may influence interconnected neuromuscular, cerebrovascular, cardiopulmonary, metabolic, and inflammatory domains. Available evidence indicates that immobility is associated with progressive disruption of neural, vascular, and metabolic coordination, whereas early mobilization may provide structured physiological input that supports partial re-engagement of regulatory mechanisms when applied within individual tolerance limits. Low- to moderate-intensity activity has been proposed, based on clinical and mechanistic evidence, to involve pathways related to neurovascular coupling, mitochondrial bioenergetics, and activity-dependent neuroplasticity; however, direct patient-level evidence in acute neurocritical care remains limited. Physiological stability during mobilization should therefore be interpreted as a dynamic process rather than a binary condition, with transient hemodynamic, intracranial, and respiratory fluctuations requiring contextual assessment. Interpretation is further limited by methodological heterogeneity, variable outcome definitions, and differences in patient severity and monitoring approaches. Integrated monitoring and data-informed decision support may help identify patient-specific tolerance thresholds. Overall, early mobilization is best understood as a context-dependent, threshold-sensitive intervention requiring continuous system-level assessment.

## 1 Introduction

Immobility in neurocritical care extends beyond the absence of movement and is associated with early, measurable alterations in neuromuscular function, cerebral perfusion, and systemic physiological regulation (1). A reduction in mechanical loading initiates early alterations in skeletal muscle homeostasis, characterized by diminished motor unit recruitment, suppression of anabolic signaling, and activation of proteolytic pathways. These effects extend beyond peripheral tissues (2). Concurrent changes emerge in cerebral perfusion, autonomic regulation, and microvascular responsiveness, particularly in the context of acute neurological injury, where central control mechanisms are already compromised (3). Early mobilization represents a controlled re-engagement of these disrupted inputs that normally coordinate neural input, metabolic demand, and vascular adaptation (4). For this narrative review, early mobilization is defined as a structured, clinician-supervised, and progressively graded physical activity intervention initiated during the acute neurocritical care phase once minimum physiological safety criteria are met (5–7). This includes passive and active range-of-motion exercises, bed mobility, sitting, standing, transfer training, assisted ambulation, passive verticalization/head-up tilt positioning, and device-assisted mobilization, but excludes routine repositioning alone, unsupervised activity, and rehabilitation delivered exclusively in chronic or post-acute settings (7–10). In this manuscript, a neurocritically ill patient is defined as an adult patient with an acute neurological or neurosurgical condition requiring intensive or intermediate neurocritical care monitoring because of potential or ongoing disturbances in consciousness, cerebral perfusion, intracranial dynamics, systemic hemodynamics, respiratory function, or neurological stability (6, 11–13). This definition includes patients with conditions such as acute ischemic or hemorrhagic stroke, traumatic brain injury, subarachnoid hemorrhage, intracranial hemorrhage, severe encephalopathy, status epilepticus, central nervous system infection, and postoperative neurosurgical states, while excluding pediatric populations, chronic stable neurological disorders, and long-term rehabilitation populations (6, 11–13). Within this framework, immobility functions as an active driver of system-level disorganization rather than a passive consequence of critical illness (14, 15). Its defining feature depends not only on intensity, but also on timing and modulation of activation. This establishes a regulated balance between physiological activation and system stability (16). Clinically, this balance is often reflected in variable tolerance to even low-intensity activity during early mobilization. Rather than a simple intervention, early mobilization introduces controlled physiological stress to systems that are already unstable and functionally impaired. Mechanical activation may support muscle preservation, regional perfusion, and adaptive signaling responses; however, it may also transiently challenge cardiovascular stability and neurological integrity (17). The overall effect is likely shaped by the interaction between intervention characteristics and patient-specific physiological reserve, including intracranial dynamics, depth of sedation, and hemodynamic capacity (18). Mechanistically, this balance is interpreted here as potentially involving the coupling between mechanical stimuli and intracellular signaling networks (19). Evidence from experimental, rehabilitation, and non-neurocritical settings indicates that contractile activity and mechanical stretch can influence pathways involved in protein synthesis, mitochondrial function, cytoskeletal organization, endothelial behavior, and tissue perfusion (19). In acute neurocritical populations, however, these pathways should be regarded as biologically plausible rather than directly demonstrated mechanisms of early mobilization. Early mobilization may help re-engage aspects of these signaling pathways, but the activation thresholds required to produce consistent biological effects remain uncertain (20). Evidence from controlled settings suggests that even low-intensity inputs can influence key regulatory processes, yet translation to clinically heterogeneous neurocritical populations remains limited (21). Clinical implementation is constrained by competing priorities and the need for continuous risk assessment. Neurological instability, fluctuating systemic parameters, and the presence of life-sustaining therapies frequently necessitate cautious progression (22). Current monitoring frameworks capture only partial system behavior and provide incomplete representations of tolerance, often reflecting selected physiological variables without fully representing integrated system responses (22, 23). As a result, mobilization strategies often rely on clinical judgment, introducing variability across practitioners and care environments (23). Methodological limitations further shape interpretation. Differences in intervention protocols, outcome definitions, patient populations, monitoring strategies, adverse-event definitions, and assessment timing reduce comparability across studies (6, 24). Functional recovery is also influenced by baseline physiological reserve, injury type, disease severity, the duration of prior immobility, and the timing and intensity of mobilization (25, 26). Accordingly, associations between mobilization and outcomes require cautious interpretation because interacting clinical and physiological factors limit causal inference, particularly in heterogeneous neurocritical and mechanically ventilated ICU populations (24, 27). Although early mobilization has been discussed in relation to safety, functional recovery, cerebral perfusion, systemic hemodynamics, neuromuscular decline, respiratory complications, metabolic–inflammatory regulation, and neurological recovery, these domains are often examined separately (6, 28). This fragmentation leaves an important knowledge gap regarding how timing, intensity, physiological reserve, and tolerance thresholds interact during acute neurocritical care (26, 29). Consistent with the operational definitions provided above, this review is restricted to structured, clinically supervised mobilization strategies initiated during the acute phase of adult neurocritical care and excludes pediatric populations, chronic stable neurological disorders, long-term rehabilitation settings, and highly specialized interventions outside routine clinical practice (27, 28). The body of this review uses a thematic narrative synthesis to integrate mechanistic and clinical evidence across neuromuscular, cerebrovascular, cardiopulmonary, metabolic, inflammatory, neurological recovery, safety, monitoring, and implementation domains (25, 29). The main objective of this narrative review is to clarify how early mobilization may influence neurological recovery and physiological stability in adult neurocritical care by interpreting it as a threshold-regulated and context-dependent intervention for individualized clinical decision-making.

## 2 Methods

This narrative review was developed using a non-systematic literature search and thematic synthesis approach. The initial search was conducted between January and March 2026 using PubMed/MEDLINE, Embase, Scopus, Web of Science, the Cochrane Library, and Google Scholar. A supplementary search was conducted between April and June 2026 using the same databases and search concepts. The combined searches covered publications available from database inception through June 2026. Foundational older studies were retained when necessary to support core concepts related to cerebral autoregulation, intracranial pressure, cerebral perfusion pressure, neurovascular coupling, ICU-acquired weakness, critical care rehabilitation, and early mobilization safety. Search terms addressed early mobilization and rehabilitation, neurocritical and intensive care, acute neurological injury, cerebral and systemic hemodynamics, ICU-acquired weakness, neuromuscular and respiratory assessment, physiotherapy, mobilization readiness, and mobilization safety. Terms were adapted to the indexing and syntax of each database.

Sources were included when they addressed adult neurocritical or critically ill populations and examined early mobilization or rehabilitation, physiological safety, neurological or functional recovery, ICU-acquired weakness, cerebral or systemic hemodynamics, respiratory function, bedside monitoring, adverse events, or implementation in intensive care settings. Priority was given to human clinical studies, guidelines, consensus statements, systematic reviews, meta-analyses, and clinically relevant mechanistic evidence. Sources focused exclusively on pediatric populations, stable chronic neurological disorders, outpatient or long-term rehabilitation without an acute critical care context, non-clinical exercise physiology without direct relevance to neurocritical care, or interventions outside routine ICU mobilization practice were excluded. Evidence from these settings was not used to support clinical recommendations, physiological thresholds, safety criteria, or demonstrated effects in adult neurocritical care. Mechanistic evidence from outside the acute neurocritical setting was retained only when directly applicable clinical evidence was unavailable and was identified as indirect or hypothesis-generating.

Additional sources were identified through backward and forward citation tracking. Each citation was checked against the specific statement it supported, considering the population, clinical setting, study design, outcome, and scope of inference. References that did not directly support the accompanying claim or were inconsistent with the eligibility criteria were removed or replaced. Duplicate records were consolidated before the reference list was updated and renumbered. To improve the credibility and transparency of the narrative synthesis, the manuscript was reviewed with reference to relevant Joanna Briggs Institute principles for narrative textual evidence, with particular attention to source credibility, logical consistency, citation directness, distinction between direct and indirect evidence, and acknowledgment of evidentiary limitations.

Because this article was designed as a narrative review, no formal PRISMA-based screening process, pooled analysis, or study-level risk-of-bias assessment was performed. Reference selection was guided by relevance to the review objective, clinical applicability, methodological quality, and contribution to the interpretation of early mobilization as a threshold-regulated intervention in adult neurocritical care.

## 3 Consequences of immobility in neurocritical illness

### 3.1 Neuromuscular decline and ICU-acquired weakness

Neuromuscular decline in neurocritical illness reflects a progressive disruption in the coupling between neural activation and muscle maintenance, rather than a delayed consequence of inactivity alone (30, 31). Reduced central drive, often influenced by sedation and impaired cortical signaling, leads to early attenuation of motor unit recruitment and destabilization of neuromuscular junction function (30, 31). At the same time, the absence of mechanical loading shifts intracellular regulation toward net protein loss, with suppression of anabolic signaling and activation of proteolytic pathways (32). These changes are accompanied by declining mitochondrial efficiency and reduced oxidative capacity, limiting energy availability and impairing contractile performance. Clinically observed weakness may reflect not simply a loss of strength, but a breakdown in coordinated regulation across neural and muscular systems (33). ICU-acquired weakness can be understood as a system-level outcome shaped by interacting neural, metabolic, and structural disturbances (32, 33). Muscle integrity depends on coordinated neural and mechanical input. In intact systems, this coordination sustains protein turnover, mitochondrial function, and cytoskeletal stability (34). When neural activation is diminished, this alignment becomes disrupted, leading to a gradual loss of functional coherence across multiple levels of organization (35). However, the trajectory of decline is not uniform. Variability in baseline physiological reserve, depth of sedation, inflammatory burden, and duration of immobilization introduces marked heterogeneity, limiting the predictability of progression and response (36). Direct identification of underlying mechanisms at the bedside remains limited. Standard assessments capture global strength or electrophysiological patterns but do not reliably distinguish between reversible functional impairment and established structural damage (36).

#### 3.1.1 Bedside diagnostic criteria for ICU-acquired weakness

For methodological clarity, ICU-acquired weakness should be linked to standardized bedside diagnostic criteria rather than described only as a mechanistic consequence of immobility (37, 38). In cooperative patients, the Medical Research Council sum score (MRC-SS) remains the most widely used clinical tool for identifying ICU-acquired weakness (39). The MRC-SS evaluates six bilateral muscle groups, with each muscle graded from 0 to 5, producing a total score from 0 to 60 (40). A score below 48/60 is commonly used to identify clinically relevant ICU-acquired weakness, whereas lower scores, particularly below 36/60, may indicate more severe weakness (40, 41). However, this assessment requires adequate arousal, comprehension, and cooperation, which limits its applicability in deeply sedated, delirious, aphasic, or severely encephalopathic neurocritical patients (37, 42). Handgrip dynamometry provides a simple quantitative adjunct for assessing peripheral strength when the patient can follow commands (43). Commonly used thresholds for clinically relevant weakness include grip strength below 11 kg in men and below 7 kg in women, although interpretation should account for age, sex, dominant hand, premorbid function, focal paresis, and upper-limb injury (44). When bedside strength testing is not feasible or when the neuromuscular substrate of weakness is unclear, nerve conduction studies and electromyography provide complementary diagnostic information (41, 45). Nerve conduction studies can help identify critical illness polyneuropathy through reduced compound muscle action potentials and sensory nerve action potential abnormalities, whereas electromyography can support the identification of critical illness myopathy or combined polyneuromyopathy (45, 46). These tools are particularly relevant in neurocritical care because diffuse ICU-acquired weakness must be distinguished from focal neurological deficits caused by the primary brain injury (Table 1) (42, 46).

**Table 1:** Standardized bedside and electrophysiological tools for identifying ICU-acquired weakness.

Assessment tool	What it evaluates	Diagnostic value	Key limitation	References
Medical Research Council Sum Score (MRC-SS)	Voluntary strength in six bilateral muscle groups; total score 0–60	MRC-SS < 48/60 supports ICU-acquired weakness; <36/60 may indicate severe weakness	Requires wakefulness, comprehension, and cooperation	(40)
Handgrip dynamometry	Quantitative grip strength	Grip strength <11 kg in men or <7 kg in women supports clinically relevant weakness	Limited by consciousness, cooperation, hand dominance, focal paresis, and upper-limb injury	(44)
Nerve conduction studies	Peripheral nerve function and compound/sensory action potentials	Helps identify critical illness polyneuropathy and axonal involvement	Requires equipment and expertise; may be difficult during unstable ICU care	(47)
Electromyography	Muscle membrane excitability and motor unit morphology	Helps differentiate critical illness myopathy from neuropathic weakness	Interpretation may be limited by edema, sedation, cooperation, and technical constraints	(47, 48)
Combined clinical and electrophysiological assessment	Strength, peripheral nerve function, and muscle substrate	Improves differentiation of diffuse ICU-acquired weakness from focal neurological deficits	Not always feasible as routine bedside screening	(49)

#### 3.1.2 Bedside ultrasound assessment of muscle architecture

Beyond clinical strength testing and electrophysiological assessment, this limitation should also be interpreted in light of contemporary advances in non-invasive bedside imaging. Point-of-care ultrasound has become an increasingly important tool for serial assessment of peripheral muscle architecture in critically ill patients, particularly for detecting ICU-acquired weakness and early skeletal muscle deterioration (50, 51). Measurements of muscle thickness, cross-sectional area, echogenicity, and pennation characteristics, particularly in muscles such as the rectus femoris, vastus intermedius, and quadriceps group, can provide real-time information on early muscle wasting or atrophy, edema, fatty infiltration, and qualitative architectural change before overt weakness becomes clinically apparent (51, 52). In addition, diaphragm ultrasound can assess respiratory muscle structure and function through parameters such as diaphragm thickness, thickening fraction, and excursion, thereby linking peripheral neuromuscular decline with respiratory muscle dysfunction and ventilator liberation outcomes (53, 54). These approaches do not directly identify intracellular or ultrastructural mechanisms of ICU-acquired weakness, but they substantially improve bedside detection and longitudinal monitoring of muscle deterioration (50, 52). In this context, ultrasound-based monitoring may help distinguish evolving structural changes from purely functional limitation during critical illness and recovery from mechanical ventilation (54). In addition, much of the mechanistic framework is derived from controlled or non-neurocritical settings, where the interaction between neurological injury and systemic stress is less pronounced (55). Accordingly, observed weakness may reflect overlapping functional and structural contributions rather than a single definable process. At the bedside, this may manifest as disproportionate fatigue or reduced responsiveness during minimal voluntary movement.

### 3.2 Cerebral perfusion and autoregulatory sensitivity

#### 3.2.1 Vulnerability to positional and hemodynamic shifts

Cerebral perfusion in neurocritical illness is governed by a dynamic interaction between systemic hemodynamics and intrinsic autoregulatory mechanisms that act to stabilize cerebral blood flow across fluctuating pressures (56). Under normal conditions, autoregulation maintains relatively stable perfusion despite moderate variations in mean arterial pressure through coordinated vascular responses (56, 57). In the setting of neurological injury, however, this regulatory capacity becomes variably impaired, shifting the brain toward greater dependence on external hemodynamic stability (57). As a result, even modest positional adjustments or changes in systemic pressure may produce disproportionate alterations in cerebral blood flow. Clinically, such responses may be observed during head elevation or early sitting, where small positional changes are associated with measurable hemodynamic variability. Apparent transient instability may reflect a narrowed operational range within which cerebral perfusion can be maintained, although this may vary across patients (58). This increased sensitivity results from combined disturbances in vascular reactivity and in the coupling between perfusion, metabolic demand, and neural activity (58). An essential regulatory feature is the pressure–flow relationship that defines the limits of autoregulation. When this relationship becomes shifted or compressed, tolerance to hemodynamic variation decreases, rendering cerebral perfusion more susceptible to routine clinical interventions (59). Positional changes, including head elevation or early mobilization, influence venous outflow, intracranial pressure, and arterial inflow. Their net effect is not uniform and depends on both the integrity and temporal variability of autoregulatory responses (60). This variability introduces a degree of uncertainty into otherwise standardized care practices. Continuous bedside characterization of autoregulatory status is incomplete. Current monitoring approaches provide indirect or intermittent estimates and may fail to capture rapid or region-specific fluctuations in perfusion (61). Moreover, much of the mechanistic understanding is derived from controlled or simplified conditions that do not fully reflect the complexity of acute neurocritical illness (62, 63). Consequently, apparent physiological stability may mask a reduced tolerance to otherwise routine hemodynamic variation, particularly when autoregulatory reserve is impaired after acute brain injury (58–63).

#### 3.2.2 Implications for perfusion heterogeneity

Cerebral perfusion heterogeneity in neurocritical illness reflects an uneven spatial distribution of blood flow across brain regions, arising from the interaction between impaired autoregulation and regionally variable vascular responsiveness. Under physiologically intact conditions, regional cerebral blood flow is matched to local metabolic demand through coordinated interactions among neurons, astrocytes, pericytes, endothelial cells, and vascular smooth muscle (64, 65). In the presence of neurological injury, this coordination becomes disrupted. Local differences in vascular tone, microvascular integrity, and tissue metabolism give rise to divergent perfusion patterns (66), in which some regions may receive adequate flow while others remain relatively underperfused (66). These spatial disparities do not follow a uniform trajectory; rather, they fluctuate in response to both local tissue conditions and systemic influences. Variations in arterial pressure, carbon dioxide levels, and oxygen delivery may further amplify these imbalances, increasing the degree of mismatch between perfusion and metabolic demand (67). An important regulatory component relates to the regional variability of autoregulatory thresholds. When autoregulation is partially preserved in some areas but impaired in others, global hemodynamic adjustments can produce uneven effects across the cerebral circulation (68). Interventions that increase systemic pressure may improve perfusion in pressure-dependent regions while exposing relatively intact areas to excessive flow (69). Similarly, positional changes or modifications in ventilatory settings may redistribute perfusion in ways that are difficult to anticipate (56). The pattern is therefore not uniform hypoperfusion, but may reflect spatial imbalance in blood flow and metabolic support (61). Available monitoring approaches predominantly capture global or averaged perfusion signals, which may obscure clinically relevant regional differences (70). Even when advanced modalities are employed, their temporal or spatial resolution may be insufficient to detect dynamic shifts in perfusion distribution (70). As a consequence, region-specific vulnerability may remain unrecognized despite apparently adequate global perfusion.

### 3.3 Cardiopulmonary deconditioning

Cardiopulmonary deconditioning in neurocritical illness develops through a progressive uncoupling between metabolic demand and the capacity of the cardiovascular and respiratory systems to deliver and utilize oxygen (71). Reduced physical activity leads to diminished venous return, decreased stroke volume, and reduced cardiac efficiency, while prolonged recumbency alters vascular tone and promotes redistribution of blood volume (72). In parallel, respiratory function is affected by decreased diaphragmatic activity and reduced chest wall movement, resulting in impaired ventilation and less effective gas exchange (73). These effects emerge through interacting physiological pathways rather than isolated mechanisms and are further shaped by autonomic dysregulation and inflammatory signaling, contributing to a decline in overall physiological reserve (73, 74). Reduced exercise tolerance often reflects a broader limitation in integrated oxygen transport and utilization across systems, which may become evident even during low-intensity mobilization (74). Coordination between cardiac output, vascular tone, and ventilatory drive determines how the system adapts to rising metabolic demand. These elements normally adjust dynamically to meet fluctuations in metabolic demand (75). With immobility, however, this coordination weakens. Baroreflex sensitivity may decline, peripheral vascular responses become less adaptive, and ventilatory patterns lose efficiency (76). As a result, even minor increases in activity can lead to disproportionate physiological strain. At the bedside, this may present as early tachycardia, increased respiratory effort, or rapid onset of fatigue during low-intensity mobilization. The degree of deconditioning varies according to baseline physiological reserve and duration of immobilization. Baseline cardiovascular and respiratory reserve, duration of immobilization, neurological severity, mechanical ventilation, autonomic dysfunction, and exposure to sedation or vasoactive support influence cardiopulmonary deconditioning and individual tolerance to early mobilization (77, 78). Clinical parameters provide only partial insight into system-level performance. Subclinical limitations in oxygen delivery or utilization may remain undetected until functional demand increases. Consequently, physiological strain may emerge before overt signs of instability become apparent (79). These responses may precede overt clinical instability.

### 3.4 Metabolic and inflammatory alterations

Metabolic and inflammatory alterations in neurocritical illness arise from the combined effects of acute neurological injury, systemic stress, organ-support therapies, and prolonged physical inactivity (49, 80). Critical illness promotes catabolic signaling, accelerated muscle protein breakdown, mitochondrial dysfunction, impaired glucose utilization, insulin resistance, and altered inflammatory activity (81). These responses vary according to injury type, illness severity, nutritional status, inflammatory burden, organ dysfunction, and duration of immobilization. Metabolic instability should therefore be understood as a dynamic interaction among substrate availability, energy demand, mitochondrial capacity, endocrine regulation, and immune activation rather than as a single isolated disturbance (49, 80–83). This interaction involves the integration of metabolic demand with inflammatory signaling. When regulatory coupling is preserved, these systems operate in coordination, allowing energy production, substrate utilization, and immune responses to remain balanced (84). In the context of immobility, this coordination becomes disrupted. Reduced muscle activity limits glucose uptake and oxidative metabolism, while inflammatory mediators may further impair mitochondrial function and promote insulin resistance (80, 85). This state is marked by reduced metabolic flexibility and inefficient energy utilization (86, 87). These metabolic alterations may influence tolerance to early mobilization, as even low levels of activity require adaptive responses that are not fully preserved (88, 89). Interpretation of metabolic and inflammatory status based on available indicators remains limited. Common biomarkers provide partial or time-limited information and may not reflect dynamic interactions between pathways. As a result, underlying metabolic constraints during early activity may be underestimated (90, 91). This integrated disruption across neuromuscular, cerebrovascular, cardiopulmonary, and metabolic–inflammatory domains is summarized in Figure 1.

**Figure 1:**
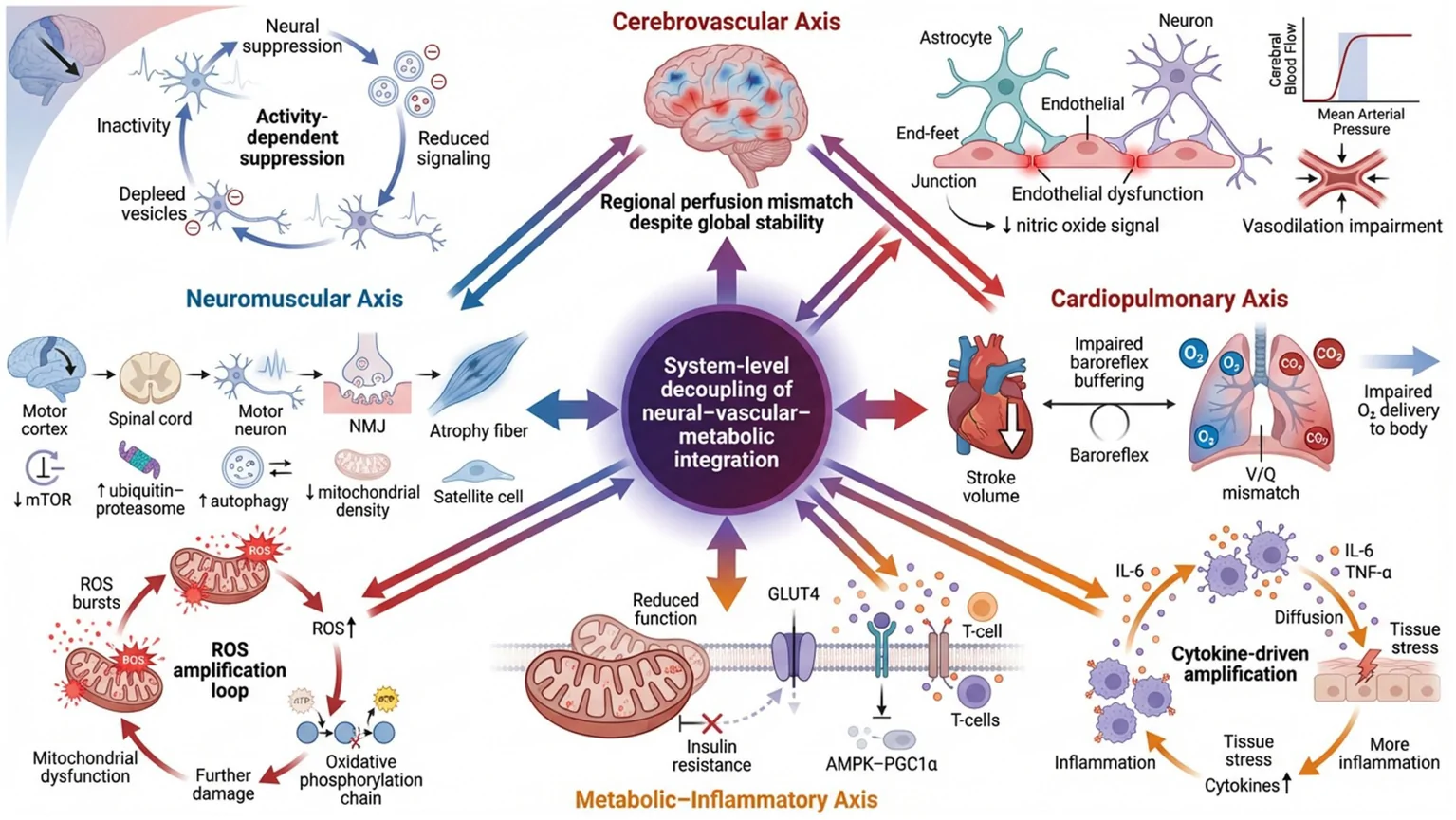
System-level decoupling across neuromuscular, vascular, and metabolic axes.

Immobility initiates a distributed disruption in neural–vascular–metabolic coordination, illustrated by convergent alterations across interconnected physiological axes. Within the neuromuscular domain, reduced central drive and neuromuscular junction activity coincide with suppression of mTOR signaling, activation of ubiquitin–proteasome pathways, and mitochondrial decline, collectively favoring atrophy. Concurrently, cerebrovascular regulation shows endothelial dysfunction, reduced nitric oxide signaling, and impaired vasodilation, contributing to regional perfusion mismatch despite preserved global flow. Cardiopulmonary adaptation is characterized by attenuated baroreflex buffering, reduced stroke volume, and ventilation–perfusion mismatch, limiting effective oxygen delivery. In parallel, metabolic–inflammatory signaling involves AMPK–PGC1α modulation, GLUT4 dysregulation, and cytokine-driven amplification loops (e.g., IL-6, TNF-α), with ROS-mediated feedback reinforcing mitochondrial dysfunction. Arrows denote directional interactions, while amplification loops represent self-reinforcing processes rather than linear causality. This conceptual synthesis is supported by evidence on ICU-acquired weakness, muscle wasting, cerebrovascular dysregulation, cardiopulmonary deconditioning, metabolic dysfunction, and inflammatory signaling during critical illness and immobility (92–95).

## 4 Mechanistic basis of early mobilization

### 4.1 Neurovascular coupling

Neurovascular coupling underlies the capacity of the brain to match regional blood flow to local metabolic demand through coordinated interactions between neurons, glial cells, and the vascular endothelium (96). Under baseline conditions, neuronal activation initiates signaling cascades that lead to vasodilation, enabling targeted delivery of oxygen and substrates to active regions. In neurocritical illness, this coordination is frequently disrupted (96, 97). Injury-related alterations in endothelial function, astrocytic signaling, and microvascular integrity weaken the relationship between neural activity and perfusion (98). As a result, increases in metabolic demand may not be matched by adequate blood flow, while in other regions, perfusion may become decoupled from actual tissue requirements. This mismatch may contribute to localized vulnerability and may influence the trajectory of neurological recovery (99). Early mobilization may introduce controlled changes in systemic and neural activation that interact with these disrupted pathways. Increased afferent input, modest elevations in metabolic demand, and shifts in hemodynamic state are hypothesized to engage residual neurovascular responses, although this engagement varies across patients and is shaped by injury severity, microvascular integrity, and overall systemic stability (100). Any re-establishment of functional coupling during mobilization should therefore be interpreted as partial, context-dependent, and not directly demonstrable using routine bedside monitoring. In some patients, mobilization may coincide with more effective distribution of cerebral blood flow, whereas in others it may reveal regions where regulatory mechanisms remain insufficient to maintain balance between demand and supply (101). Current assessment approaches rely on indirect or global indicators of perfusion rather than direct measurement of coupling dynamics. These methods often fail to capture regional variability or rapid temporal changes. Consequently, localized mismatches between perfusion and metabolic demand may persist despite apparently stable systemic conditions (101, 102). Accordingly, mobilization effects are interpreted here as potentially involving partial recoupling across systems rather than as evidence of a directly demonstrated pathway. The proposed mechanistic sequence linking afferent activation to microvascular responses within the neurovascular unit is depicted in Figure 2.

**Figure 2:**
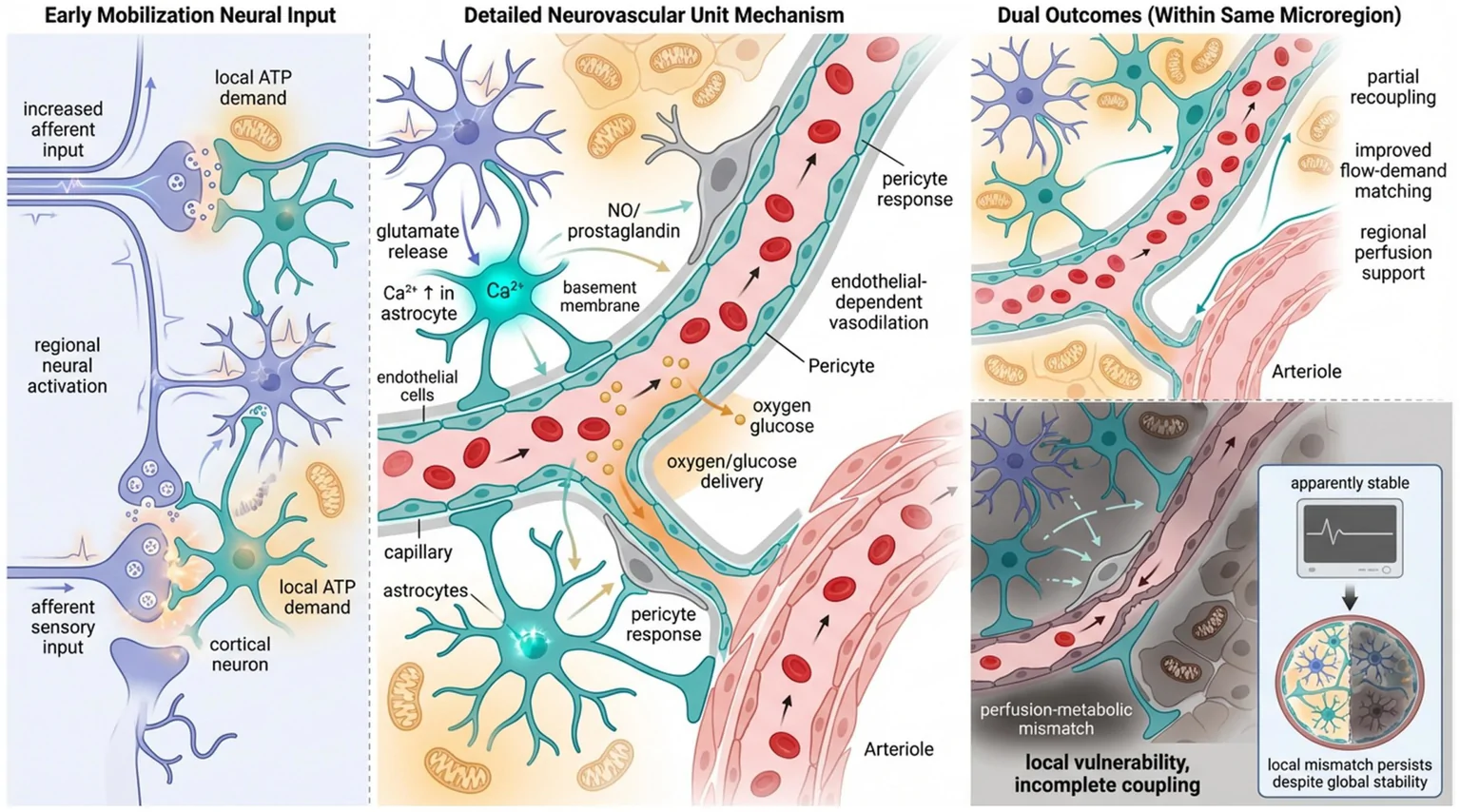
Neurovascular coupling responses to mobilization-derived neural input.

Early mobilization introduces afferent-driven neural activation that increases local ATP demand and initiates signaling within the neurovascular unit. Neuronal glutamate release elevates astrocytic Ca^2+^, which propagates toward vascular interfaces and promotes production of nitric oxide and prostaglandins, acting on endothelial cells and pericytes to regulate capillary tone. This cascade represents a proposed pathway through which endothelial-dependent vasodilation and oxygen–glucose delivery may be supported within the neurovascular unit. The depicted arrows indicate directional flow from neural activation to vascular response, while pericyte-mediated modulation represents a locally regulated control node rather than a uniform response. In the same microregion, outcomes diverge: partial recoupling may improve flow–demand matching, whereas incomplete signaling leads to perfusion–metabolic mismatch despite stable global indices, indicating context-dependent restoration of coupling. This figure integrates evidence on neurovascular coupling, endothelial and pericyte-mediated vascular responses, impaired coupling after acute brain injury, and the limited translation of these mechanisms to mobilization in neurocritical populations (11, 100, 103–105).

### 4.2 Systemic hemodynamic adaptation

Systemic hemodynamic adaptation during early mobilization reflects the capacity of the cardiovascular system to redistribute blood flow in response to changing physiological demands. Under resting conditions, cardiac output, vascular tone, and venous return are dynamically coordinated to maintain systemic perfusion. In critically ill patients, this coordination may be disrupted by autonomic dysfunction, sedation, positive-pressure ventilation, intravascular volume disturbance, vasoactive therapy, and prolonged recumbency (106, 107). In neurocritical illness, this balance is frequently altered by autonomic dysregulation, shifts in intravascular volume, and the effects of sedation or vasoactive support (108). When mobilization is initiated, even at low intensity, these systems are required to adjust rapidly. Increases in venous return, redistribution of blood volume, and changes in vascular resistance must occur in coordination to sustain adequate perfusion (108, 109). Here, what appears as hemodynamic instability may reflect a reduced capacity to accommodate transitions between passive and active physiological states, rather than representing immediate physiological failure (110). A primary mechanism involves the interaction between cardiac output regulation and peripheral vascular responsiveness. Baroreflex function plays a key role in buffering acute changes in blood pressure, while vascular tone adjusts to maintain effective circulation (111). In the setting of immobility and neurological injury, these responses may be attenuated or delayed (20). As a result, mobilization can produce transient reductions or fluctuations in blood pressure, often accompanied by compensatory increases in heart rate (112). Clinically, these changes may be observed during transitions from supine to upright positioning or during assisted standing. The magnitude and pattern of these responses vary across individuals. Baseline cardiovascular reserve, volume status, autonomic function, duration of immobilization, sedation exposure, mechanical ventilation, and vasoactive support influence the capacity to adapt safely to postural transitions and activity-related physiological demand (77, 78). Standard bedside measurements provide only a partial representation of these regulatory dynamics. Parameters such as heart rate and arterial pressure reflect global responses but do not fully capture regional perfusion changes or short-term adaptive processes (2). Consequently, transient hemodynamic variability observed during mobilization may represent ongoing physiological adjustment rather than inherently adverse events (16).

### 4.3 Skeletal muscle activation and metabolic signaling

Skeletal muscle activation during early mobilization represents a plausible interface through which mechanical input may influence metabolic and intracellular signaling responses. Under conditions of immobility, reduced contractile activity limits the stimuli required to sustain protein synthesis, mitochondrial function, and glucose uptake (113). This shift may promote proteolysis, impair oxidative metabolism, and contribute to reduced insulin sensitivity. With the reintroduction of even low-intensity movement, contractile activity may reactivate aspects of signaling pathways involved in energy regulation and cellular maintenance, although direct confirmation of these intracellular responses during acute neurocritical mobilization remains limited (114). The magnitude of this response depends on prior deconditioning and neuromuscular integrity, as well as substrate availability and the effectiveness of neural activation (115, 116). The regulatory framework underlying these processes may depend on the coupling between mechanical load and metabolic signaling. In stable physiological states, muscle contraction coordinates substrate utilization with energy demand, maintaining metabolic flexibility (117). Under neurocritical conditions, this coordination becomes unstable and frequently decoupled (117). Reduced neural drive and prolonged inactivity diminish the responsiveness of key signaling pathways, limiting the efficiency of metabolic adaptation. As a result, even modest activation may lead to disproportionate metabolic strain or incomplete functional recovery (118). Variability in inflammatory status and systemic stress further modulates these responses, influencing how muscle tissue responds to mobilization (119). Direct evaluation of intracellular metabolic signaling is not feasible in routine clinical practice. Available indicators, including circulating biomarkers and global performance measures, provide indirect insight and may not accurately reflect localized cellular activity (119, 120). Nevertheless, ultrasound-derived measures may serve as clinically accessible structural and functional surrogates of neuromuscular and metabolic deterioration during immobilization and reactivation (50, 121). Serial changes in muscle thickness, cross-sectional area, echogenicity, and elastography-derived stiffness may reflect the cumulative consequences of altered proteostasis, edema, and muscle quality during critical illness (121, 122). Diaphragm ultrasound may identify reduced respiratory muscle contractile activity during prolonged ventilation or limited mobilization through measures such as diaphragm thickness, thickening fraction, and excursion (123). Thus, although point-of-care ultrasound does not measure intracellular signaling pathways directly, it provides a practical bedside bridge between mechanistic concepts and observable tissue-level adaptation during early mobilization (122, 123). Consequently, engagement of signaling pathways may remain insufficient to support full functional adaptation despite apparent physiological activation. The molecular signaling modules linking mechanical activation to metabolic adaptation are summarized in Supplementary Table S1.

### 4.4 Interaction with inflammatory pathways

The interaction between early mobilization and inflammatory pathways reflects a bidirectional relationship in which mechanical activity influences immune signaling, while inflammatory status shapes physiological responsiveness to movement (124). In neurocritical illness, immobility is commonly associated with a sustained low-grade inflammatory state characterized by altered cytokine profiles and immune cell activation (125). This state may extend beyond the primary site of neurological injury, affecting peripheral tissues and contributing to metabolic and vascular dysfunction (126). With the introduction of controlled movement, mechanical stimuli may modulate inflammatory signaling through changes in circulation, tissue perfusion, and cellular stress responses. These effects depend on baseline inflammatory burden and systemic stability (77). However, direct mobilization-specific evidence for these inflammatory pathways in acute neurocritical populations remains limited (12, 127). Their direction and magnitude are further shaped by intervention timing and overall physiological condition. A key regulatory feature involves the interaction between inflammatory mediators and cellular pathways involved in adaptation and repair (128). In the absence of pathological stress, transient inflammatory responses support tissue remodeling and recovery. In the context of prolonged inactivity and critical illness, this balance may shift toward sustained or dysregulated activation (129). Early mobilization may influence this balance by altering the distribution and clearance of inflammatory mediators and by engaging pathways linked to oxidative metabolism and cellular resilience (130). At the same time, elevated inflammatory activity may limit the capacity of tissues to respond effectively to mechanical input, creating a feedback loop between reduced adaptability and persistent signaling disturbance (131). Circulating markers provide limited resolution of local inflammatory dynamics and temporal variability (132). Systemic measurements may therefore fail to capture tissue-specific responses to mobilization, particularly in heterogeneous clinical conditions. The threshold-dependent interaction between mobilization inputs and inflammatory–metabolic responses within an integrated physiological network is illustrated in Figure 3. The cross-domain physiological responses and their context-dependent constraints are summarized in Table 2.

**Figure 3:**
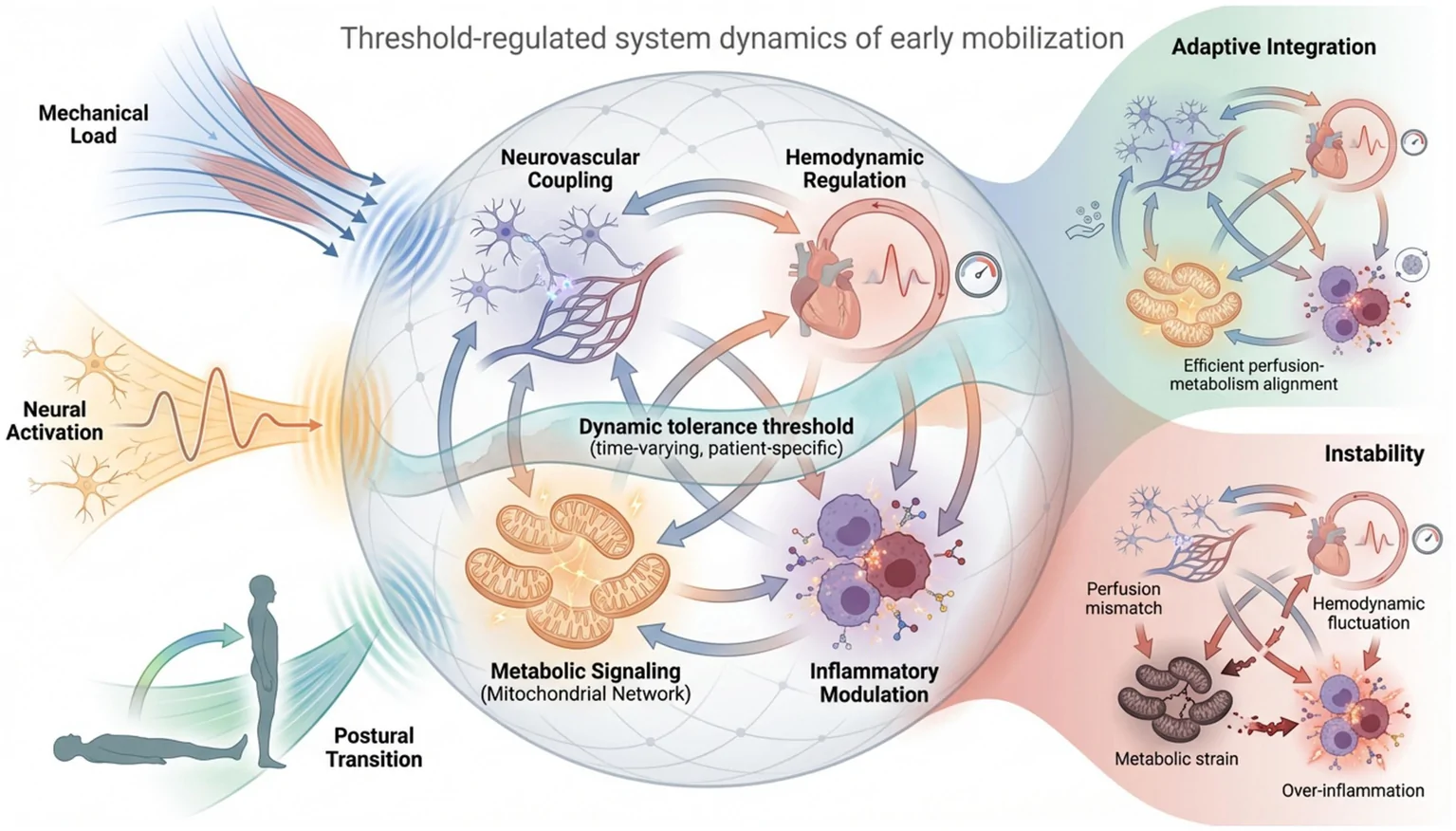
Threshold-governed integration across neurovascular and inflammatory axes.

**Table 2:** System-level responses, constraints, and bedside monitoring signals during early mobilization across physiological domains.

Domain	Baseline disruption (immobility)	Mobilization input	Adaptive response	Constraint/failure mode	Clinical/bedside monitoring signal	References
Neuromuscular	Reduced motor unit recruitment, proteolysis, and early deterioration of muscle architecture	Low-load activation	Partial restoration of protein synthesis signaling and neuromuscular engagement	Inadequate neural drive → incomplete activation; progressive muscle wasting or altered muscle quality may be under-recognized without serial assessment	Weakness despite activity; reduced muscle thickness or cross-sectional area; increased echogenicity or altered pennation pattern on point-of-care muscle ultrasound	(50, 53, 133)
Cerebrovascular	Impaired autoregulation and perfusion mismatch	Postural + metabolic demand shifts	Partial neurovascular recoupling	Narrow autoregulatory range → instability	Fluctuating CPP/flow	(134)
Hemodynamic	Reduced baroreflex and volume redistribution	Orthostatic + activity-induced demand	Compensatory HR and vascular tone adjustments	Delayed or insufficient buffering	BP variability and tachycardia	(135)
Respiratory	Reduced diaphragm activity, V/Q mismatch, and respiratory muscle deconditioning	Increased ventilatory demand	Increased respiratory drive and partial recruitment of respiratory muscle activity	Inefficient ventilation, early fatigue, or impaired diaphragm contractile response	Rapid shallow breathing; reduced diaphragm excursion or reduced diaphragm thickening fraction on diaphragm ultrasound	(136–138)
Metabolic	Reduced oxidative capacity and insulin resistance	Contractile metabolic signaling	Partial mitochondrial activation	Limited substrate utilization	Early fatigue and low activity tolerance	(88, 92, 139)
Inflammatory	Low-grade chronic activation	Circulatory + mechanical modulation	Redistribution/partial modulation	Persistent dysregulation	Variable inflammatory markers	(140)

Early mobilization is depicted as a convergent input integrating mechanical load, neural activation, and postural transition within a coupled physiological network. Neurovascular coupling, hemodynamic regulation, mitochondrial metabolic signaling, and inflammatory modulation interact bidirectionally around a dynamic tolerance threshold that varies over time and between patients. Directional arrows indicate cross-axis propagation rather than linear causality, with feedback loops reflecting reciprocal modulation between perfusion, metabolism, and immune signaling. Within this bounded range, coordinated responses support partial recoupling and improved alignment between oxygen delivery and metabolic demand. When system responses extend beyond this threshold, the same pathways are associated with hemodynamic fluctuation, perfusion mismatch, metabolic strain, and amplified inflammatory activity. This framework represents a systems-level model of context-dependent adaptation rather than a fixed-response mechanism. This conceptual framework is supported by evidence on neurovascular coupling, hemodynamic adaptation, skeletal muscle metabolic signaling, inflammatory modulation, and ICU mobilization tolerance, while the proposed cross-domain interactions remain hypothesis-generating in neurocritical populations (11, 29, 92, 141).

## 5 Neurological recovery

### 5.1 Activity-dependent neuroplasticity

Activity-dependent neuroplasticity reflects the capacity of the nervous system to reorganize in response to patterned activation, linking functional recovery to the quality and timing of neural input (142). Where normal control mechanisms remain functional, repeated activation strengthens synaptic connections, supports dendritic remodeling, and promotes adaptive refinement of neural circuits (143, 144). In neurocritical illness, this capacity is variably disrupted by structural injury, altered neurotransmission, and reduced afferent and efferent signaling (145). Immobility further limits the sensory and motor inputs required to sustain these processes, contributing to a decline in network responsiveness (146). Early mobilization reintroduces structured sensory and motor input that may provide conditions compatible with activity-dependent neural adaptation. However, direct evidence that acute ICU mobilization produces measurable neuroplastic reorganization in neurocritical patients remains limited (147, 148). The biological rationale for activity-dependent plasticity is based on the principle that the timing, repetition, salience, specificity, and intensity of neural activity can influence synaptic and structural adaptation after brain injury (149, 150). Repetitive, task-relevant input can influence calcium-dependent signaling, gene expression, and structural remodeling within neural circuits. In the context of neurological injury, these pathways may remain partially functional but operate within constrained limits (150). The effect of mobilization depends on how the intensity and pattern of activity align with the remaining functional capacity of neural networks to respond (151). In clinical settings, this may correspond to variability in motor response or delayed improvement despite repeated activation. In some cases, graded activation may support stabilization of functional pathways. In others, insufficient or poorly timed input may produce limited effects or transient disruption (152). Functional changes may not directly correspond to underlying circuit adaptation, particularly in early phases of recovery (153). Clinical indicators often lag behind biological processes, and variability in injury severity further complicates interpretation. Much of the current understanding is derived from controlled or non-critical settings, where confounding influences are minimized (154). Therefore, the relevance of activity-dependent neuroplasticity to early mobilization in acute neurocritical care should be interpreted as mechanistically plausible rather than directly demonstrated unless supported by patient-level neurocritical evidence (12, 127, 147).

#### 5.1.1 Synaptic responsiveness and functional reorganization

Synaptic responsiveness and functional reorganization may represent core processes through which neural circuits adapt to changing patterns of activity, linking cellular signaling to measurable aspects of neurological recovery (142, 150, 151, 155). In unperturbed biological conditions, synaptic strength is continuously modulated through activity-dependent mechanisms that regulate neurotransmitter release, receptor sensitivity, and network connectivity (156). In neurocritical illness, these processes are disrupted by structural injury, altered excitability, and reduced afferent input. Immobility further limits the patterned stimulation required to sustain synaptic adaptation, contributing to decreased responsiveness and impaired integration across neural circuits (157). Early mobilization reintroduces graded sensory and motor input that may engage residual synaptic plasticity, allowing partial reorganization of functional pathways rather than restoration of pre-injury states (156, 157). At the mechanistic level, this depends on the balance between large-scale network connectivity within neural networks. This balance determines the capacity of synapses to respond effectively to incoming stimuli and to participate in coordinated activity (158). Following neurological injury, shifts in this equilibrium may reduce signal fidelity or produce unstable patterns of activation. The effect of mobilization, therefore, depends on how patterned input interacts with this altered synaptic environment (159). In some cases, repeated activation may support stabilization of functional connectivity. In others, the same input may produce limited integration or transient dysregulation, depending on the integrity of local circuits and the timing of intervention (160). Functional integration may progress unevenly, with periods of apparent stability interspersed with reduced synaptic responsiveness (161). Available clinical measures do not capture synaptic-level processes and may overlook subtle adaptations occurring within neural networks. Accordingly, synaptic responsiveness and functional reorganization are discussed here as inferred mechanisms that may contribute to recovery, not as established effects of early mobilization in acute neurocritical patients (12, 127).

### 5.2 Delirium and cognitive trajectories

Delirium and cognitive trajectories reflect alterations in network-level organization that extend beyond the limits of focal structural injury, encompassing disruptions that affect attention, arousal regulation, and executive processing (162). When system-level coordination is maintained, cognitive function depends on coordinated activity across distributed neural networks, a coordination that is sustained by stable neurochemical signaling and adequate cerebral perfusion (162). Within the context of critical illness, however, this coordinated organization becomes progressively unstable. A range of factors, including systemic inflammation, impaired neurotransmission (162, 163), disruption of normal sleep architecture, and fluctuating hemodynamic conditions, contribute to the emergence of both transient and persistent disturbances in cognitive processing (163). Immobility may further reduce the degree of sensory input and environmental engagement, thereby reinforcing disorganization within these large-scale neural networks (164). Early mobilization introduces a structured form of external stimulation that can interact with these processes, potentially promoting wakefulness, enhancing the integration of sensory inputs, and modulating arousal states in a manner that may partially counterbalance ongoing disruption, although effects may vary across clinical contexts (165). Clinically, this may be reflected in transient improvements in alertness or engagement during and immediately after mobilization sessions. This process depends on the balance between large-scale network connectivity and neuromodulatory control mechanisms. Systems that govern attention and arousal depend on coordinated interactions between cortical and subcortical regions, interactions that remain sensitive to both internal physiological states and externally driven stimuli (166, 167). In neurocritical illness, this balance may shift toward reduced connectivity or toward patterns of signaling that become unstable over time (167). The influence of mobilization is therefore contingent on how externally applied activation aligns with these altered network states. In certain contexts, graded and appropriately timed activity may support more coherent engagement across networks and contribute to stabilization of cognitive function (168). In other contexts, particularly when physiological instability persists, excessive or poorly timed stimulation may instead exacerbate existing disorganization rather than mitigate it (169). Cognitive patterns may fluctuate over time without providing a direct or reliable reflection of underlying network recovery, particularly during the early phases of illness (170). Clinical assessment tools primarily capture behavioral manifestations of cognitive state, whereas the neural dynamics that underlie these observations may evolve along different temporal trajectories and may not be fully represented within standard clinical measures. Experimental findings indicate that even low-intensity activation may engage residual regulatory pathways. The divergence of cognitive trajectories under mobilization-induced network modulation is illustrated in Figure 4.

**Figure 4:**
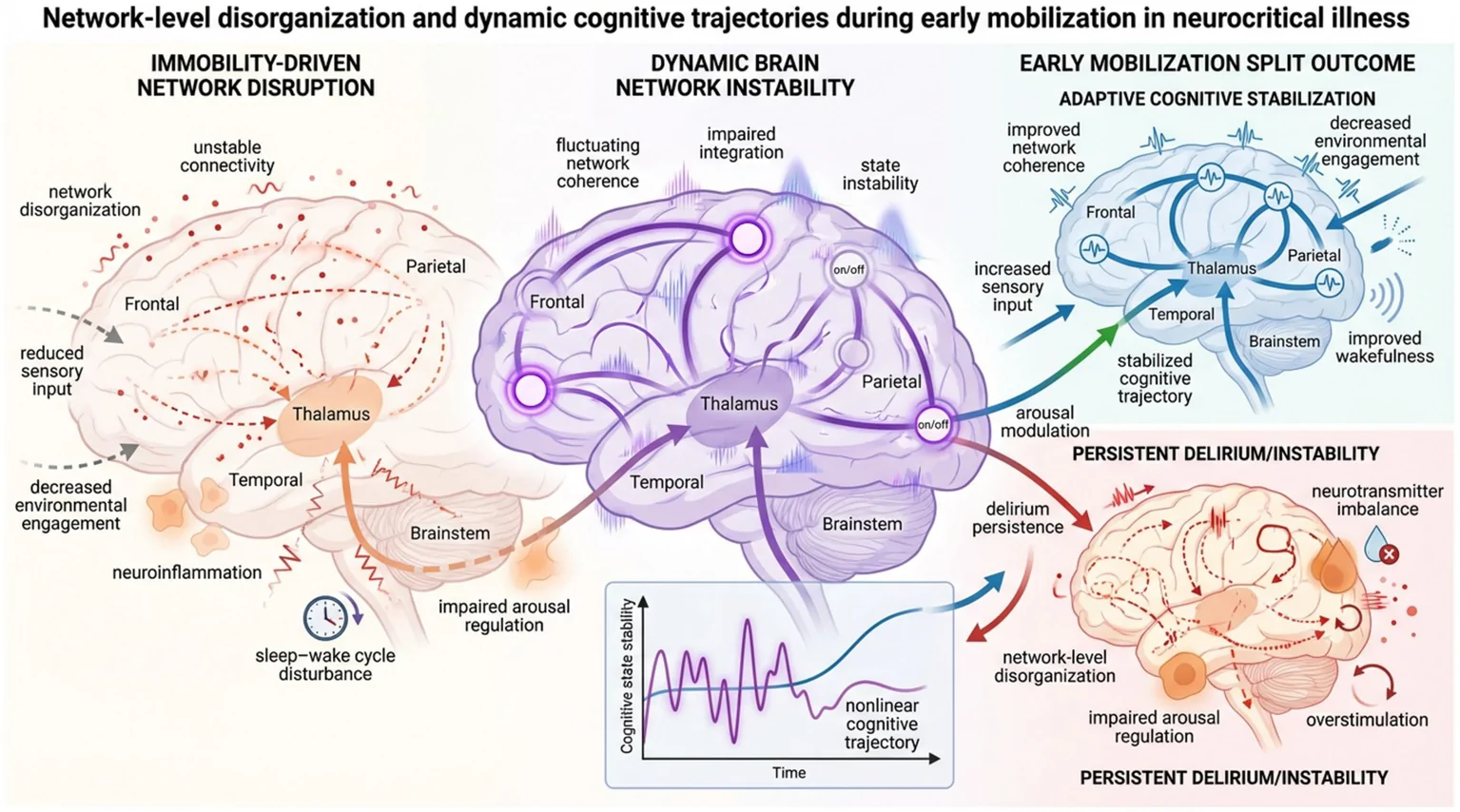
Network instability and divergent cognitive trajectories during mobilization.

Immobility is associated with reduced sensory input and environmental engagement, contributing to disrupted connectivity across frontal, parietal, temporal, and thalamic networks, alongside impaired arousal regulation and sleep–wake disturbance. These alterations propagate into a state of dynamic network instability, characterized by fluctuating coherence and impaired integration across large-scale circuits involving the thalamus and brainstem. Early mobilization introduces structured sensory input and arousal modulation that interact with these unstable networks. Within the same microregional context, outcomes diverge: one trajectory reflects a hypothesized improvement in network coherence and cognitive stability, whereas the alternative pathway shows persistent disorganization, neurotransmitter imbalance, and delirium persistence. The depicted transitions represent nonlinear and state-dependent processes rather than uniform recovery pathways. This figure is based on evidence linking critical illness, inflammation, impaired neurotransmission, sleep–wake disruption, sensory deprivation, delirium, and early mobilization-related arousal effects, while the depicted network trajectories remain conceptual rather than directly demonstrated pathways (171–174).

### 5.3 Timing across injury types and pathology-specific vulnerability windows

The timing of early mobilization across different types of neurological injury reflects a critical interaction between the evolving pathophysiology of neural damage and the capacity of the patient to tolerate externally imposed activation (175, 176). This relationship is not uniform across neurocritical diagnoses. Acute ischemic stroke, hemorrhagic stroke, aneurysmal subarachnoid hemorrhage, traumatic brain injury, and diffuse encephalopathic states differ in their dominant windows of vulnerability (6, 11, 175, 176), including perfusion dependence, risk of hemorrhagic expansion, intracranial compliance, vasospasm, metabolic instability, and impaired arousal regulation (6, 11). Accordingly, mobilization timing should not be interpreted only as “early” or “delayed,” but as a dynamic decision aligned with pathology-specific biological constraints (177). In acute ischemic stroke, the earliest phase is shaped by the vulnerability of the ischemic penumbra, impaired autoregulation, and dependence on adequate cerebral perfusion. Mobilization within the first 24–48 h may therefore require particular caution, especially when neurological deficits are fluctuating, reperfusion status is uncertain, or systemic blood pressure changes could compromise collateral flow (7, 178). In hemorrhagic stroke, the dominant early constraint is the risk of hematoma expansion, mass effect, and intracranial pressure instability; therefore, mobilization should be considered only after neurological and hemodynamic stability have been established, and there is no evidence of ongoing expansion or deterioration (6, 179). In aneurysmal subarachnoid hemorrhage, timing depends strongly on aneurysm status and vasospasm risk. Before aneurysm securing, mobilization is limited by the risk of rebleeding and abrupt hemodynamic stress. After clipping or coiling, the risk profile changes, but delayed cerebral ischemia and vasospasm, particularly during the typical vasospasm window, remain major constraints that may narrow the safe mobilization range (7, 11, 178). In traumatic brain injury, the key biological constraint is reduced intracranial compliance and the potential for mobilization-induced changes in intracranial pressure, cerebral perfusion pressure, and autoregulatory reserve. Even low-intensity activity or postural transition may be poorly tolerated when intracranial pressure is unstable, cerebral perfusion pressure is marginal, or sedation and ventilatory support are required to maintain physiological control (10, 180). In diffuse encephalopathy, status epilepticus, central nervous system infection, or postoperative neurosurgical states, mobilization readiness is shaped less by a single lesion-specific threshold and more by the interaction between arousal, seizure control, systemic inflammation, respiratory reserve, and metabolic stability (181, 182). Thus, the relevant vulnerability window is defined by the recovery of coordinated multisystem responsiveness rather than by elapsed time alone. These pathology-specific considerations indicate that early mobilization should be guided by dynamic thresholds rather than fixed timelines (4). Relevant thresholds include stability of neurological examination, absence of progressive intracranial or hemorrhagic deterioration, preservation of cerebral perfusion, controlled systemic hemodynamics, adequate oxygenation and ventilatory reserve, and the ability to tolerate postural transition without sustained physiological decompensation (177, 183). The major injury-specific constraints and mobilization windows are summarized in Table 3.

**Table 3:** Pathology-specific vulnerability windows and dynamic thresholds for early mobilization in neurocritical care.

Neurocritical condition	Vulnerability window	Dominant constraint	Dynamic threshold before mobilization/progression	Mobilization implication	References
Acute ischemic stroke	First 24 h; penumbral/reperfusion phase	Penumbral viability, collateral-flow dependence, impaired autoregulation	Stable or improving neurological status; BP within individualized perfusion targets; no deterioration with head-up positioning	Avoid rapid or prolonged mobilization during unstable perfusion; begin low-intensity activity after stabilization	(20)
Intracerebral hemorrhage/non-aneurysmal hemorrhage	First 24–48 h; hematoma expansion phase	Hematoma expansion, perihematomal edema, mass effect, ICP risk	Controlled BP; stable neurological examination; no evidence of expansion or raised ICP	Delay or limit mobilization during active expansion risk; progress with graded bedside-to-upright transitions	(184)
Aneurysmal SAH before securing	Pre-clipping/coiling phase	Rebleeding risk, BP surges, hydrocephalus, ICP instability	Aneurysm status confirmed; BP controlled; ICP/EVD stable; no acute deterioration	Restrict to essential positioning and passive activity; avoid abrupt hemodynamic stress	(109)
Aneurysmal SAH after clipping/coiling	Vasospasm/DCI window	Vasospasm, delayed cerebral ischemia, and CPP vulnerability	Aneurysm secured; CPP/ICP stable; no worsening vasospasm or DCI; tolerated postural change	Progress cautiously according to perfusion status, vasospasm surveillance, and neurological response	(185)
Traumatic brain injury	Acute low-compliance phase	Restricted intracranial compliance, ICP/CPP instability, impaired autoregulation	ICP controlled; CPP maintained; no ICP rise or CPP drop with head elevation; stable ventilation	Defer or restrict mobilization if ICP/CPP unstable; use stepwise postural progression under monitoring.	(56)
Diffuse encephalopathy/CNS infection/status epilepticus	Variable systemic–neurological instability phase	Impaired arousal, seizure risk, inflammation, metabolic instability	Seizures controlled; arousal improving; temperature/metabolic status stable; adequate oxygenation	Base readiness on multisystem responsiveness, not time alone; start with low-stimulation, low-intensity activity	(182)
Postoperative neurosurgical states	Immediate postoperative phase	Surgical-site stability, edema, CSF dynamics, drain/EVD constraints	Stable neurological status; no surgical complication; CSF/ICP stable; drain safety confirmed	Follow neurosurgical clearance; progress according to surgical, neurological, and physiological stability	(11)

### 5.4 Limits of causal interpretation

The causal interpretation of observational early-mobilization studies is limited by non-random treatment allocation, time-varying illness severity, intervention timing and dose, and changes in the physiological condition of the patient over the course of critical illness (186). Improvements observed in neurological or functional outcomes are frequently attributed to mobilization; however, such associations emerge within clinical environments in which multiple variables are simultaneously changing (187). Patients who receive mobilization may differ systematically from those in whom mobilization is delayed or withheld because of neurological instability, sedation, mechanical ventilation, vasopressor requirements, or poorer baseline function, thereby creating confounding by indication and selection bias (188, 189). When eligibility, treatment assignment, and the beginning of follow-up are not aligned, observational analyses may also be affected by immortal-time and other time-dependent biases (190). A central issue lies in the interplay between selection, tolerance, and response. Patients who can receive or tolerate early mobilization may differ systematically from those who are not, reflecting differences in illness severity, sedation level, organ support, vasopressor exposure, baseline functional reserve, and physiological resilience (187). At the same time, the response to mobilization itself is shaped by the same constraints described in earlier sections, including limitations related to metabolic capacity, hemodynamic stability, and neural integrity (191). These overlapping influences create a setting in which disentangling direct causal effects becomes methodologically challenging. Observed associations between mobilization and outcome may therefore reflect the alignment of recovery trajectories rather than the presence of a discrete intervention effect (191, 192). Additional variability in study design, definitions of outcome, and timing of assessment further complicates interpretation and limits comparability across studies.

### 5.5 Structured synthesis of primary clinical evidence

To help readers distinguish between different levels of clinical evidence, we added a structured synthesis of key interventional and observational studies that directly inform early mobilization in neurocritical or closely related critically ill populations. This table summarizes study design, population, sample size, mobilization exposure or protocol, comparator condition, principal outcomes, safety findings, and a brief methodological-quality note. The purpose of this synthesis is not to replace a systematic review or formal risk-of-bias assessment, but to make the evidentiary basis of the narrative review more transparent and to prevent findings from randomized trials, observational cohorts, pilot studies, and systematic reviews from being interpreted with equivalent certainty (Table 4).

**Table 4:** Structured synthesis of key clinical evidence underpinning early mobilization in neurocritical and critically ill populations.

Study	Design	Population/setting	Sample size	Mobilization protocol/exposure	Comparator or reference condition	Main outcomes	Safety findings	Methodological-quality note	Relevance to the present framework	References
AVERT Trial Collaboration Group, 2015	Multicenter randomized controlled trial	Adults with acute stroke treated within 24 h of symptom onset	*n* = 2,104	Very early mobilization initiated within 24 h, with frequent out-of-bed activity	Usual stroke-unit care	Very early, higher-dose mobilization reduced the odds of a favorable functional outcome at 3 months compared with usual care.	Safety outcomes were systematically monitored; the findings raised concern regarding excessive timing and dose rather than establishing that all early mobilization is unsafe.	Large, high-quality multicenter RCT; strong evidence for timing- and dose-related caution, although conducted mainly in stroke-unit rather than neuro-ICU populations.	Provides direct evidence that earlier and more intensive mobilization is not universally beneficial and supports threshold-regulated dosing.	(193)
Langhorne et al., 2018	Cochrane systematic review of randomized controlled trials	Adults with acute stroke receiving very early or delayed mobilization	9 RCTs; *n* = 2,958	Very early mobilization, generally initiated within 24–48 h of stroke onset, with protocol-dependent frequency and intensity	Usual care or delayed mobilization	Very early mobilization did not clearly improve death or poor functional outcome; substantial uncertainty remained regarding the optimal timing, frequency, and dose.	No uniform excess of serious adverse events was demonstrated across all trials, but the intervention and safety definitions were heterogeneous.	High-level evidence synthesis; dominated numerically by AVERT and limited by heterogeneity in intervention definitions, dose, and outcome measurement.	Reinforces that stroke evidence must be interpreted according to timing and dose rather than as support for indiscriminate early escalation.	(194)
Yen et al., 2024	Single-center randomized controlled trial	Adults with moderate-to-severe traumatic brain injury in a trauma ICU	*n* = 65	Early progressive out-of-bed mobilization initiated within 7 days after injury	Early progressive upright positioning and in-bed upright rehabilitation	Early progressive out-of-bed mobilization was associated with better mobility and functional outcomes and shorter ICU stay and mechanical-ventilation duration.	Mobilization was feasible under protocolized monitoring; the modest sample size limits the precision of uncommon adverse-event estimates.	Randomized design provides stronger causal evidence than observational studies, but the small, single-center sample limits generalizability.	Supports pathology-specific progression in TBI when ICP/CPP, ventilation, consciousness, and neurological status are monitored.	(195)
Takara et al., 2024	Multicenter retrospective propensity score-matched study	Adults with aneurysmal subarachnoid hemorrhage receiving physical and/or occupational therapy at five Japanese hospitals	718 screened; *n* = 450 eligible; 229 early and 221 delayed before matching; 122 patients per group after matching	Walking training initiated within 14 days after aSAH onset	Walking training initiated after 14 days	Favorable functional outcome occurred more frequently in the matched early-mobilization group than in the delayed group: 81.1% versus 52.5%; risk difference 28.7, 95% CI 17.4–39.9.	The study did not demonstrate a clear increase in mobilization-related neurological harm among clinically selected patients.	Multicenter study with propensity-score matching; residual confounding, immortal-time considerations, and selection according to readiness for walking remain possible.	Supports cautious post-securing walking progression while emphasizing that the observed association should not be interpreted as definitive causation.	(175)
Foudhaili et al., 2024	Systematic review and meta-analysis	Adults with aneurysmal subarachnoid hemorrhage	16 studies; 4,757 participants	Early mobilization and rehabilitation strategies after aSAH	Usual care or delayed mobilization, depending on the included study	Early mobilization was associated with better functional status and a lower reported cerebral-vasospasm rate, although certainty was limited.	Available studies generally suggested feasibility and acceptable safety in appropriately selected patients.	Review-level evidence limited by predominantly observational designs, variable definitions of early mobilization, and clinical heterogeneity.	Supports aSAH-specific vulnerability windows, including aneurysm securing, vasospasm/DCI risk, hydrocephalus, and neurological stability.	(176)
Jia et al., 2025	Prospective–retrospective matched controlled study	Adults with traumatic brain injury or hypertensive intracerebral hemorrhage in neurocritical care	*n* = 58; 29 intervention and 29 matched controls	Passive head-up tilt positioning using a motorized standing bed	Standard care	The intervention group showed improved neurological-recovery measures, fewer ICU-related complications, and shorter NSICU and hospital stays.	ICP, CPP, PRx, respiratory mechanics, and neurological variables were monitored during treatment.	Clinically relevant matched comparison, but non-randomized and small; residual confounding remains possible.	Supports passive verticalization as a graded low-intensity option when active participation is not yet feasible.	(181)
Küçük et al., 2025	Prospective cohort study with repeated physiological measurements	Mechanically ventilated adult ICU patients receiving passive range-of-motion mobilization	*n* = 20	Passive range-of-motion exercises applied to the extremities with serial optic nerve sheath diameter and hemodynamic assessment	Within-patient measurements before, during, and after passive mobilization	Passive mobilization was not associated with a significant increase in optic nerve sheath diameter or clinically important hemodynamic deterioration.	No evidence of increased intracranial pressure or clinically important instability was identified during the monitored sessions, including in patients receiving vasopressor support.	Small, single-center physiological cohort; ONSD is an indirect ICP surrogate and the study was not designed to assess functional efficacy or rare adverse events.	Supports repeated intracranial and systemic monitoring and the distinction between transient physiological variation and sustained intolerance.	(179)
Schweickert et al., 2009	Two-center randomized controlled trial	Previously functionally independent adults receiving mechanical ventilation in medical ICUs	*n* = 104; 49 intervention and 55 control	Early physical and occupational therapy initiated during daily interruption of sedation	Daily interruption of sedation with therapy according to usual practice	The intervention increased return to independent functional status at hospital discharge and was associated with shorter delirium duration and more ventilator-free days.	One serious desaturation event occurred during 498 therapy sessions; no falls or accidental extubations were reported during therapy.	Landmark RCT with clinically meaningful outcomes; modest sample size, two-center design, and non-neurocritical population limit direct neuro-ICU generalizability.	Demonstrates that early, protocolized rehabilitation may improve functional recovery when integrated with sedation management and careful monitoring.	(196)
Bahouth et al., 2018	Quasi-experimental consecutive pre–post implementation study	Adults with primary intracerebral hemorrhage admitted to an academic neuroscience critical care unit	*n* = 57; 28 before and 29 after algorithm implementation	Formalized progressive NCCU mobility algorithm assigning passive or active pathways according to consciousness and motor function	Care during the 6-month period before algorithm implementation	After implementation, patients were more likely to be mobilized within the first 7 days: OR 8.7, 95% CI 2.1–36.6.	No neurological deterioration, hypotension, falls, or line dislodgements were reported in association with mobilization.	Small, single-center, non-randomized implementation study; susceptible to temporal confounding and selection bias.	Provides direct neurocritical evidence that diagnosis-specific, staged mobilization can be implemented safely in selected patients with acute ICH.	(197)
Olkowski and Shah, 2017	Narrative clinical review	Neurocritical care populations, including acute stroke, TBI, aSAH, ICH, and patients with neurological devices	Not applicable	Review of readiness screening, graded progression, physiological monitoring, device management, and multidisciplinary implementation	Not applicable	Synthesized the available rationale, barriers, and practical considerations for early mobilization in the neuro-ICU.	Emphasized neurological, hemodynamic, respiratory, intracranial, and device-related precautions rather than proposing unrestricted mobilization.	Neurocritical-specific review with strong clinical relevance, but not primary evidence and unable to establish efficacy or causal safety estimates.	Provides a direct conceptual foundation for individualized readiness assessment, graded progression, and interruption criteria in the present framework.	(198)
Maia et al., 2024	Narrative clinical review	Critically ill adults with neurological pathology	Not applicable	Synthesis of early-mobilization concepts in neurological critical illness	Not applicable	Identified feasibility considerations, barriers, potential outcomes, and implementation requirements.	Emphasized individualized assessment and cautious interpretation in neurological populations.	Useful for clinical and conceptual integration but not primary efficacy evidence.	Supports the clinical scope of neurocritical mobilization and the need for practical implementation criteria.	(6)
Nobles et al., 2024	Narrative review	Neurocritical care populations	Not applicable	Review of challenges and opportunities for neurocritical mobilization	Not applicable	Highlighted barriers involving neurological instability, invasive devices, staffing, uncertainty, and protocol heterogeneity.	Discussed diagnosis-specific and device-related safety concerns in neuro-ICU implementation.	Contemporary neurocritical-specific review; not primary interventional evidence.	Supports multidisciplinary roles, device precautions, and individualized implementation within the threshold-regulated framework.	(11)
Hodgson et al., 2022, TEAM Trial	Multicenter randomized controlled trial	Mechanically ventilated adult ICU patients	*n* = 750	Increased early active mobilization during mechanical ventilation	Usual level of mobilization	Increased early active mobilization did not improve days alive and out of hospital at 180 days.	Adverse events potentially related to mobilization occurred more often in the intervention group.	Large, high-quality multicenter RCT; strong ICU evidence but not specific to neurocritical populations.	Demonstrates that increasing mobilization intensity beyond usual care does not necessarily improve recovery and may increase adverse events.	(199)
Paton et al., 2024	Systematic review and meta-analysis of randomized controlled trials with frequentist and Bayesian analyses	Critically ill adults who required invasive mechanical ventilation during an acute ICU admission	67 RCTs; *n* = 7,004 participants; 59 trials contributed to meta-analysis; adverse-event meta-analysis: 20 trials; mortality meta-analysis: 58 trials, *n* = 6,218	Active mobilization involving patient participation, delivered during or after mechanical ventilation, with variation in timing, duration, intensity, and mobility level	No mobilization, delayed mobilization, or mobilization delivered at a lower frequency or intensity	Mobilization did not significantly increase adverse events compared with usual care (RR 1.09, 95% CI 0.69–1.74) and did not affect mortality (RR 0.98, 95% CI 0.87–1.12).	The overall adverse-event frequency was 2.96%, based on 693 events during 23,395 mobilization sessions across 25 studies. Most reported events were transient or non-serious, and subgroup analyses did not clearly identify a specific implementation variable that consistently increased harm.	Large systematic review restricted to RCTs, with frequentist and Bayesian analyses and GRADE assessment; adverse-event evidence had very low certainty and substantial heterogeneity, and incomplete reporting limited subgroup allocation and causal interpretation. Acute neurological conditions were excluded, limiting direct neurocritical generalizability.	Supports the general safety of active mobilization in mechanically ventilated adult ICU patients while emphasizing standardized adverse-event reporting, patient-specific stopping criteria, and caution when extrapolating findings to neurocritical populations.	(24)
Schaller et al., 2024	Expert-panel clinical guideline	Critically ill adult ICU patients	Not applicable	Evidence- and consensus-based recommendations for positioning and early mobilization	Not applicable	Provides practical recommendations concerning readiness, mobilization progression, dose, contraindications, and implementation.	Explicitly addresses conditions requiring caution, interruption, or deferral.	Guideline and consensus evidence rather than primary interventional evidence.	Supports operationalization of feasibility, caution, interruption, and deferral criteria.	(7)
O’Hara et al., 2024	Clinical review	Neurological critical care populations	Not applicable	Review of innovations in early mobilization and rehabilitation in neurological critical care	Not applicable	Highlighted emerging rehabilitation approaches, monitoring strategies, technologies, and integration of rehabilitation into acute neurological care.	Emphasized feasibility, individualized protocols, and neurological and systemic safety considerations.	Review-level evidence useful for implementation framing but unable to establish causal efficacy.	Supports integration of mechanistic, functional, technological, and bedside-monitoring domains.	(200)
Arzayus-Patiño et al., 2025	Exploratory review	Adult neurocritical patients	22 studies included	Mapping of early-mobilization interventions and safety evidence in adult neurocritical care	Not applicable	Synthesized feasibility, reported adverse events, and clinical outcomes across heterogeneous neurocritical populations.	Reported generally low frequencies of serious mobilization-related events but emphasized inconsistent definitions and monitoring methods.	Neurocritical-specific evidence synthesis; limited by heterogeneity and the absence of pooled causal estimates.	Supports neurocritical-specific safety thresholds and cautious interpretation of bedside physiological changes.	(12)
Hodgson et al., 2025	Clinical practice guideline	Adult ICU rehabilitation and mobilization	Not applicable	Recommendations for assessment, physical rehabilitation, mobilization progression, monitoring, and implementation	Not applicable	Provides guidance on rehabilitation delivery, progression, documentation, and outcome assessment.	Includes practical safety, readiness, and implementation considerations.	Guideline evidence; broad adult ICU scope rather than neurocritical-specific evidence.	Supports structured progression, multidisciplinary implementation, and standardized documentation.	(27)

## 6 Functional outcomes

### 6.1 Functional independence

Functional independence reflects the integrated capacity of the individual to perform essential activities through the coordinated engagement of neural, physical, and systemic functions (201). It may emerge not from isolated improvements in strength or mobility alone, but from the convergence of neurological recovery, stabilization of physiological systems, and preservation of adaptive responsiveness across multiple domains (202). Within this framework, independence depends on the ability to sustain task-specific coordination, maintain sufficient energy availability, and support cognitive control throughout functional activity (202, 203). Immobility during critical illness contributes to rapid skeletal-muscle wasting, loss of strength, reduced neuromuscular function, cardiopulmonary deconditioning, and increasing dependence in basic mobility activities (49, 81, 204). Early mobilization reintroduces structured physical and occupational activity that engages neuromuscular, cardiopulmonary, sensory, and cognitive functions; however, it should be interpreted as one component of recovery rather than as an intervention that independently restores functional autonomy (196, 199). A key consideration is the alignment between task demands and the capacity of underlying physiological systems to sustain performance (205). Activities such as bed mobility, transfers, ambulation, and self-care require coordinated motor control, muscle strength, balance, cardiovascular and respiratory reserve, arousal, and the ability to follow commands (77, 196). When these systems remain only partially recovered, performance may become inconsistent, variable across contexts, or highly dependent on effort (206). Measurable independence may therefore emerge in a non-uniform manner, even among patients who receive comparable levels of intervention (55). At the bedside, this may appear as inconsistent performance across similar tasks performed at different times. Functional outcomes are influenced by contextual factors, including environmental conditions, availability of support, and the degree of patient engagement, all of which may differ across care settings.

### 6.2 Mobility and rehabilitation trajectory

Mobility and the broader rehabilitation trajectory in neurocritical illness reflect the progressive translation of physiological capacity into sustained and meaningful functional movement over time (207). Recovery trajectories integrate both the rate and pattern of change, linking neuromuscular function, cardiovascular support, and cognitive engagement (208, 209). Early mobilization contributes to this process through repeated and graded activation, which may help maintain continuity in functional progression rather than allowing prolonged interruption (152, 210). A key issue is the interaction between initial physiological constraints and cumulative adaptation. Early stages of recovery are often characterized by limited tolerance to activity, where fatigue, hemodynamic variability, and impaired coordination restrict progression (211). As these constraints begin to shift, repeated exposure to task-oriented movement may support more stable integration of functional capacity. Recovery after critical and neurocritical illness does not follow a uniform or consistently linear trajectory (49, 212). Acute injury characteristics, neurological severity, baseline physiological reserve, duration of mechanical ventilation and immobilization, ICU-acquired weakness, complications, and responsiveness to rehabilitation contribute to substantial variation in recovery between patients (49, 212). Progression may therefore vary in both direction and stability, rather than following a predictable or linear course (213, 214). External influences, including the care environment, intensity and consistency of rehabilitation, and continuity of intervention, further shape the observed trajectory over time.

### 6.3 Variability in outcome definitions

Variability in outcome definitions in neurocritical care reflects differences in how recovery is conceptualized, operationalized, and interpreted across both clinical and research settings (215). Functional outcomes may be defined in terms of mobility, independence, cognitive performance, or composite indices that integrate multiple domains, with each approach capturing distinct dimensions of recovery (216). Differences in outcome definitions, assessment instruments, measurement timing, and baseline functional status influence how recovery is detected and quantified and limit the attribution of observed changes to a single intervention (127, 199). In the context of early mobilization, such variability introduces additional complexity into interpretation, as interventions that are similar in structure and delivery may appear to produce different effects depending on the outcome framework that is applied (217). A key issue lies in the degree of alignment between selected outcome measures and the underlying physiological processes they are intended to represent. Measures of functional performance may not fully capture changes occurring at the level of neural adaptation, metabolic efficiency, or systemic stability (55). Conversely, early physiological improvements may not immediately translate into observable gains in functional capacity, particularly when compensatory mechanisms or contextual limitations influence performance (218). Differences in outcome definition may therefore alter both the apparent direction and magnitude of observed effects across studies (36, 219). Additional variability in the selection of assessment tools, timing of evaluation, and reporting practices further complicates comparison and limits the interpretability of findings across different clinical contexts.

## 7 Physiological stability during mobilization

### 7.1 Hemodynamic tolerance

Hemodynamic tolerance reflects the capacity of the cardiovascular system to maintain effective systemic and cerebral perfusion in the presence of externally imposed changes in posture and metabolic demand (16, 73, 220). Under stable physiological conditions, transitions from supine to upright positions are buffered through coordinated adjustments in cardiac output, vascular tone, and venous return, allowing circulation to remain within functional limits (75, 221). In critically ill patients, however, this buffering capacity is frequently impaired as a result of autonomic dysfunction, instability in intravascular volume, and the effects of sedation or vasoactive pharmacological support. When mobilization is initiated, even at low levels of intensity, these systems are required to respond rapidly (30, 222). In this setting, relatively small increases in activity may provoke disproportionate changes in arterial pressure and heart rate (16, 223). The tolerance margin is often narrow and may vary substantially depending on the underlying physiological reserve. These responses do not necessarily indicate impaired tolerance, but may instead reflect a reduced physiological margin within which equilibrium can be maintained (223). Clinically, transient fluctuations in blood pressure or heart rate may occur without requiring immediate interruption of mobilization. A central determinant of hemodynamic tolerance lies in the interaction between baroreflex responsiveness and peripheral vascular adaptation. Rapid adjustments in vascular resistance and heart rate are necessary to preserve adequate perfusion during positional transitions and increasing activity levels (55, 217). When these regulatory mechanisms are blunted, delayed, or inconsistently expressed, transient hypotension or oscillatory patterns in hemodynamic variables may emerge (108, 221). The magnitude and pattern of these responses vary across individuals and are influenced by baseline cardiovascular status, duration of immobility, and the extent of neurological impairment (108). Observable hemodynamic parameters provide only a partial representation of the underlying regulatory processes. Measurements such as blood pressure and heart rate reflect global responses but may not adequately capture regional perfusion adequacy or short-term compensatory adjustments occurring within the microcirculation (17, 108). As a result, transient fluctuations may occur without sustained compromise, while subtle forms of instability may remain undetected through standard monitoring approaches.

### 7.2 Intracranial pressure and cerebral perfusion

Intracranial pressure and cerebral perfusion during mobilization reflect a tightly coupled and dynamically evolving interaction between cranial compliance, venous outflow, and systemic hemodynamics, with cerebral perfusion pressure representing the operational consequence of these relationships (112). In the absence of injury-related disruption, postural transitions are buffered through coordinated adjustments in cerebrospinal fluid distribution, vascular tone, and intracranial volume, allowing cerebral blood flow to remain relatively stable despite moderate fluctuations in systemic pressure (56). In neurocritical illness, this buffering capacity is frequently diminished. Structural injury, cerebral edema, impaired venous drainage, and altered cerebrovascular reactivity may elevate baseline intracranial pressure or limit its ability to adapt to changing conditions (60). As a result, mobilization can introduce shifts in intracranial dynamics that alter the balance between arterial inflow and venous outflow, influencing both the magnitude and temporal stability of cerebral perfusion (224). A central determinant lies in the interaction between mean arterial pressure and intracranial pressure, which together define cerebral perfusion pressure as a dynamic variable rather than a fixed value (61). During positional change or graded activity, fluctuations in systemic pressure may occur concurrently with changes in intracranial pressure, producing variable and sometimes unpredictable effects on perfusion (225). These responses are not always synchronized. Arterial pressure may adjust rapidly through autonomic mechanisms, whereas intracranial responses may be delayed, buffered, or regionally heterogeneous depending on local compliance and vascular characteristics (226). This temporal mismatch may generate transient variability in cerebral perfusion pressure, even when averaged values appear stable over time. When autoregulatory mechanisms remain intact, compensatory vascular responses may buffer these fluctuations within a tolerable range (227). When autoregulation is impaired, however, even modest deviations may reduce effective perfusion or expose periods of localized vulnerability at the tissue level. Interpretation of intracranial and perfusion dynamics during mobilization remains constrained by the limited resolution of currently available monitoring approaches (68, 226, 227). Measurements of intracranial pressure or derived indices of perfusion are often intermittent, averaged across time, or reflective of global conditions, thereby potentially obscuring transient or region-specific variability (68). Furthermore, monitoring modalities may not fully capture the spatial heterogeneity of cerebral perfusion or the rapid temporal changes that occur during positional transitions and activity (61, 228). Consequently, recorded values may remain within acceptable ranges while localized perfusion is compromised, or transient deviations may occur without sustained impact on tissue viability. The interaction between MAP–ICP dynamics and regionally heterogeneous cerebral perfusion during mobilization is illustrated in Figure 5.

**Figure 5:**
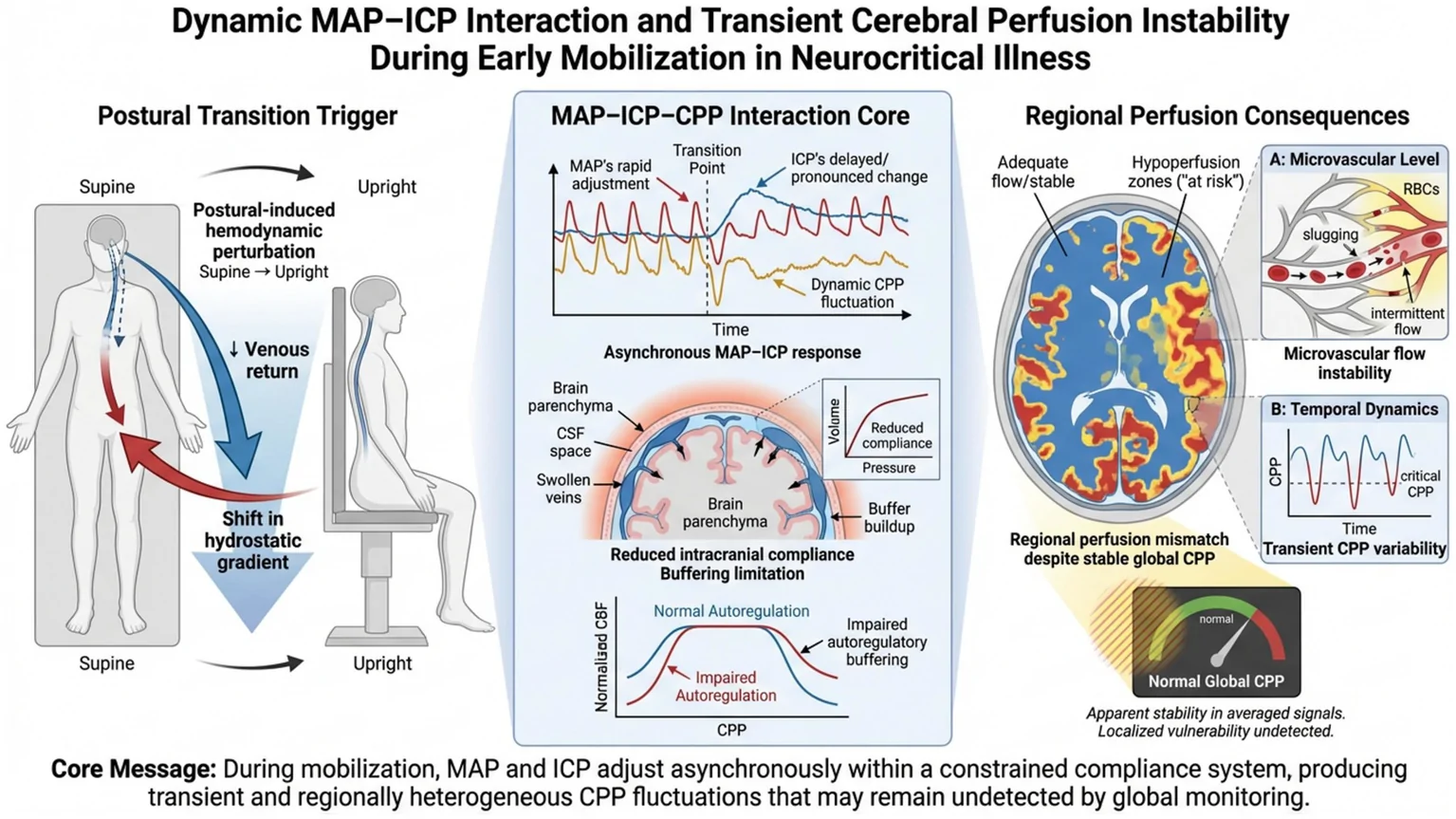
Asynchronous MAP–ICP dynamics and regional perfusion vulnerability.

Postural transition during mobilization induces a hydrostatic shift that reduces venous return and perturbs systemic pressure, initiating rapid MAP adjustment alongside delayed or amplified ICP responses within a constrained compliance system. This temporal dissociation generates dynamic CPP variability, as illustrated by non-synchronous MAP–ICP oscillations and limited intracranial buffering capacity influenced by parenchymal swelling and reduced CSF space. Although autoregulatory mechanisms may partially compensate, impaired buffering narrows the pressure–flow operating range. Downstream, these interactions produce spatially heterogeneous perfusion, where regions of hypoperfusion coexist with apparently adequate global CPP. Microvascular instability, including intermittent flow and erythrocyte sludging, further contributes to localized mismatch. The depicted relationships indicate transient, region-specific vulnerability that may remain obscured by averaged monitoring signals. This figure integrates evidence from adult acute brain injury concerning MAP–ICP interactions, CPP variability, cerebral autoregulation, intracranial compliance, venous outflow, and regional perfusion heterogeneity (229, 230).

The regulatory interactions governing perfusion stability and their monitoring constraints are summarized in Table 5.

**Table 5:** MAP–ICP coupling and perfusion stability constraints during mobilization.

Regulatory interaction axis	Mobilization-induced disturbance	Temporal coordination pattern	Observable clinical signal	Monitoring limitation	Boundary of instability	References
MAP–ICP coupling (CPP determinant)	Simultaneous fluctuation in arterial pressure and intracranial pressure	Asynchronous adjustment between MAP and ICP	Apparent stable CPP with transient variability	Intermittent ICP and averaged CPP values	Reduced CPP during transient mismatch	(225)
Cerebral autoregulation integrity	Pressure-dependent perfusion shifts	Compensatory vs. impaired buffering response	Stable systemic pressure with variable perfusion adequacy	Lack of direct autoregulatory measurement	Loss of perfusion buffering capacity	(56)
Cranial compliance–volume buffering	Postural redistribution of CSF and blood volume	Delayed intracranial pressure adaptation	Subtle ICP fluctuation during position change	Limited sensitivity to rapid changes	Exceeding compensatory reserve → ICP rise	(231)
Arterial inflow–venous outflow balance	Altered venous drainage with mobilization	Region-specific and time-variable response	Fluctuating cerebral perfusion patterns	Global metrics mask regional differences	Local congestion or hypoperfusion	(232)
Regional perfusion heterogeneity	Uneven vascular responsiveness	Spatially divergent adaptation	Normal global indices with focal vulnerability	Low spatial resolution of the monitoring	Undetected regional ischemic risk	(233)

### 7.3 Respiratory response

Respiratory response during mobilization in neurocritical illness reflects the capacity of the ventilatory system to adapt to increasing metabolic demand while maintaining effective gas exchange and airway stability (112, 234). Under physiological conditions, transitions from rest to activity are accompanied by coordinated increases in tidal volume, respiratory rate, and ventilation–perfusion matching, enabling efficient oxygen delivery and carbon dioxide clearance. In critically ill patients, this coordination is frequently disrupted (11). Reduced diaphragmatic activity, altered chest wall mechanics, and the effects of sedation or mechanical ventilation may limit ventilatory responsiveness (11, 234). As mobilization is introduced, even low-intensity activity may increase respiratory demand beyond the system’s adaptive capacity, leading to rapid, shallow breathing patterns or inefficient ventilation (181). A central determinant of respiratory tolerance lies in the interaction between ventilatory drive, respiratory muscle function, and gas exchange efficiency. Neural control of breathing must integrate metabolic signals with mechanical output, while respiratory muscles generate the force required to sustain adequate ventilation (6, 198). In neurocritical illness, this integration may be impaired due to central neurological injury, neuromuscular weakness, or disrupted coordination between inspiratory and expiratory phases (6). As a result, increases in activity may be associated with disproportionate increases in respiratory effort without corresponding improvements in gas exchange. The magnitude of this response varies across individuals and is influenced by baseline respiratory function, duration of immobility, and the level of ventilatory support (198). Interpretation of respiratory response during mobilization is further limited by the specificity of available monitoring parameters. Measurements such as respiratory rate, oxygen saturation, and end-tidal carbon dioxide provide global indicators but may not fully reflect regional ventilation–perfusion mismatch or the early development of respiratory fatigue (6, 235). Consequently, apparent stability in monitored values may coexist with inefficient ventilation, while early signs of physiological strain may remain undetected (235). Because respiratory tolerance cannot be interpreted in isolation, respiratory responses during mobilization should be integrated with neurological, intracranial, hemodynamic, metabolic, functional, and device-related signals when determining whether activity should proceed, be modified, or be stopped (7, 236). Respiratory tolerance should therefore be assessed dynamically across successive stages of mobilization rather than inferred from a single resting measurement. Progression should be modified or interrupted when increasing activity produces sustained respiratory distress, worsening gas exchange, excessive ventilatory demand, or evidence of respiratory muscle fatigue (237, 238).

## 8 Implementation and protocol considerations

### 8.1 Initiation timing and progression

The initiation of mobilization in neurocritical care represents a critical clinical decision point that reflects the balance between physiological readiness and the potential risks associated with premature activation (239). Instead of being defined by fixed or time-based protocols, the decision to initiate mobilization depends on the integration of multiple factors, including hemodynamic stability, neurological status, and the capacity of underlying regulatory systems to tolerate externally imposed demand (240). Early introduction of activity may engage adaptive mechanisms that support recovery; however, when applied during periods of instability, it may expose limited physiological reserve and precipitate disproportionate responses (239, 240). For this reason, initiation is more appropriately conceptualized as a threshold-based transition, in which readiness is determined not by elapsed time, but by the ability of interacting physiological systems to sustain controlled perturbation without loss of stability (20). Progression of mobilization extends this principle by translating initial activation into a structured and incremental increase in functional demand. This progression does not follow a strictly linear or predetermined trajectory (19). Instead, it reflects a dynamic and adaptive process shaped by continuous feedback between physiological response and imposed activity. Increases in mobilization intensity, duration, or task complexity should remain aligned with the patient’s evolving neurological, cardiovascular, respiratory, and neuromuscular capacity and should be guided by repeated bedside assessment of readiness and tolerance (11, 77, 127). When this alignment is achieved, progression may support consolidation and reinforcement of functional gains, although responses may vary across individuals. In contrast, when activity exceeds the adaptive capacity of these systems, it may result in transient instability, excessive fatigue, or regression in performance (241). The trajectory of progression, therefore, varies across individuals and is influenced by injury characteristics, baseline physiological reserve, and the interaction between intrinsic recovery processes and externally applied interventions (241). Determining appropriate timing for initiation and progression remains constrained by the absence of direct markers that reflect system-level readiness. Observable clinical parameters, including vital signs and immediate functional responses, provide only partial insight into underlying physiological stability (6). These indicators may fail to capture latent constraints or delayed responses that become apparent only with increasing activity (6). As a consequence, decisions regarding progression often rely on iterative and continuous assessment, in which adjustments are guided by patterns of tolerance and response rather than adherence to predefined or rigid protocols. In practice, this is reflected in stepwise adjustment of activity levels based on real-time physiological response rather than fixed progression criteria (29). To make progression clinically operational, early mobilization should be structured as a hierarchical activity spectrum rather than as a single intervention. The patient should first be screened against physiological safety thresholds, including neurological stability, hemodynamic tolerance, oxygenation, ventilatory support, ICP/CPP status when relevant, and device safety (15, 27, 29). Once basic feasibility is confirmed, the current mobility level should be documented using an ICU-specific mobility instrument, such as the ICU Mobility Scale, the Perme ICU Mobility Score, or the Chelsea Critical Care Physical Assessment Tool (15, 16). Progression should then occur one level at a time, from in-bed activation to upright sitting, transfers, standing, and assisted ambulation, with regression or temporary interruption when clinical or physiological instability emerges. This measurement-guided hierarchy is summarized in Table 6.

**Table 6:** Measurement-guided hierarchy for early mobilization progression in neurocritical care.

Progression level	Mobilization activity	Criteria for progression	Measurement anchor	References
Level 0: Readiness screening	Physiological screening: line, drain, airway, and device safety check	Safety thresholds acceptable; no active neurological, hemodynamic, respiratory, intracranial, or device-related contraindication	Baseline ICU Mobility Scale, Perme ICU Mobility Score, or CPAx	(242)
Level 1: In-bed activation	Passive or active-assisted range of motion, bed exercises, rolling, and bridging	Stable response to minimal activity; no new neurological decline, sustained desaturation, or hemodynamic instability	IMS 0–1; baseline Perme/CPAx function	(7)
Level 2: Upright preparation	Head-up positioning, chair position in bed, and active bed mobility	Tolerates postural change without sustained BP, SpO₂, ICP/CPP, or consciousness deterioration	IMS sitting-preparation range; Perme mobility barriers	(181)
Level 3: Sitting at the edge of the bed	Supine-to-sit transition, dangling, sitting balance training	Maintains sitting with acceptable physiological response and manageable assistance	IMS sitting level; CPAx sitting balance domain	(243)
Level 4: Transfer training	Sit-to-stand, bed-to-chair transfer, supported pivot transfer	Lines/drains secured; adequate lower-limb loading; no sustained instability during transfer	IMS transfer level; Perme transfer category	(244)
Level 5: Standing and pre-gait activity	Supported standing, weight shifting, marching in place	Tolerates upright loading; respiratory and hemodynamic responses remain within caution range	IMS standing/marching level; CPAx standing domain	(245)
Level 6: Assisted ambulation	Assisted stepping or walking with staff or device support	Prior level tolerated; no worsening neurological, respiratory, hemodynamic, or intracranial response	IMS ambulation level; Perme gait/endurance categories	(28)
Level 7: Advanced ambulation	Longer-distance ambulation or reduced assistance	Stable repeated sessions; improving functional score and physiological tolerance	IMS 9–10 when applicable; rising Perme/CPAx score	(245)
Regression/stop rule	Reduce intensity, return to prior level, or stop mobilization	Sustained instability, new neurological decline, unsafe device configuration, or failure to recover after rest	Reassess IMS, Perme, or CPAx after stabilization	(7)

### 8.2 Operational anchors for threshold-regulated mobilization

To make the central construct clinically actionable, threshold-regulated mobilization should be operationalized as a multivariable bedside framework rather than as a single universal cutoff (12, 61). In practice, the threshold is defined by the interaction between baseline physiological readiness, the patient’s response to a standardized mobilization challenge, and recovery after reduction of activity or rest (4, 12). Thus, mobilization readiness and progression should be anchored to convergent signals across neurological status, intracranial dynamics, cerebral perfusion, autoregulatory reserve, regional oxygenation, systemic hemodynamics, respiratory reserve, metabolic tolerance, neuromuscular capacity, and device safety (4, 61, 225, 246). These anchors should be interpreted dynamically and according to the underlying neurocritical diagnosis, because the same absolute value may carry different implications in ischemic stroke, intracerebral hemorrhage, aneurysmal subarachnoid hemorrhage, traumatic brain injury, or diffuse encephalopathy (61, 225, 246). The practical anchors, feasibility thresholds, caution criteria, and stopping criteria for threshold-regulated mobilization are summarized in Table 7.

**Table 7:** Operational anchors, feasibility thresholds, and stopping criteria for threshold-regulated early mobilization in neurocritical care.

Domain/anchor	Practical bedside measure	Feasible/proceed	Caution/modify intensity	Defer/stop mobilization	Evidence basis/interpretation	References
Neurological stability	Serial neurological examination, level of consciousness, focal deficits, arousal, seizure activity	Stable or improving examination; no new focal decline; no uncontrolled seizure activity	Fluctuating arousal, mild transient symptoms, and agitation that resolves with rest or environmental adjustment	New neurological deterioration, worsening consciousness, uncontrolled agitation, seizure activity, or failure to recover after rest	Core clinical safety anchor across stroke, hemorrhage, TBI, SAH, encephalopathy, and postoperative states; neurological change should override isolated physiological stability	(247, 248)
Intracranial pressure	ICP monitoring when available; response to head elevation or activity	ICP stable and generally ≤20–22 mmHg; no sustained ICP rise during head elevation or low-level activity	Brief ICP rise that returns to baseline after rest, position adjustment, or reduction in activity level	Sustained ICP > 22 mmHg, repeated ICP surges, ICP rise with head-up positioning, or activity-induced intracranial decompensation	Guideline-supported anchor in severe brain injury; should be interpreted with pathology-specific goals, cranial compliance, sedation strategy, and local neurocritical care protocols	(181, 249)
Cerebral perfusion pressure	CPP derived from MAP and ICP	CPP maintained around 60–70 mmHg or individualized target; no CPP drop during mobilization	Borderline CPP requiring close monitoring or minor support adjustment	CPP < 60 mmHg, falling CPP during activity, or need for aggressive/escalating pressor support to maintain target	Global perfusion anchor; individualized targets may be required when autoregulation is impaired or CPPopt-guided management is available	(56)
Cerebral autoregulation	PRx, Mx, COx, TCD-based indices, NIRS-derived indices, or CPPopt when available	CPP maintained near the individualized autoregulatory optimum; no worsening autoregulatory index during postural transition or low-level activity	Uncertain autoregulatory status or mild worsening during activity that recovers after rest or reduced intensity	Mobilization worsens autoregulatory status, moves CPP away from the tolerated range, or produces pressure-dependent neurological/perfusion changes.	Physiological and observational support; useful for individualized threshold setting but not universally available at the bedside	(250)
Regional cerebral oxygenation	NIRS-derived rSO₂ trends or brain tissue oxygen monitoring, when available	No sustained decline from baseline; PbtO₂ generally maintained above commonly used hypoxia thresholds when monitored	Mild transient decline that rapidly recovers with rest, posture adjustment, or oxygen/ventilator optimization	Persistent regional oxygenation decline, PbtO₂ below the accepted hypoxia threshold, or failure to recover after reducing activity	Detects regional vulnerability that may be missed by global ICP/CPP; trend interpretation is more reliable than universal rSO₂ cutoffs	(251)
Systemic hemodynamics and vasoactive support	MAP, SBP, HR, rhythm, vasopressor/inotrope trend	MAP generally ≥65 mmHg and ≤110 mmHg; HR 40–130 bpm; no new arrhythmia; no recent vasopressor escalation	Mild transient BP/HR changes, low-dose stable vasoactive support, or borderline values with team approval and close monitoring	MAP <65 mmHg, SBP < 90 mmHg, SBP > 180–200 mmHg, new arrhythmia, myocardial ischemia, syncope, escalating vasoactive support, or recent dose increase	Derived from ICU mobilization safety criteria and bedside consensus frameworks, transient adaptive responses should be distinguished from sustained instability.	(12, 252)
Respiratory reserve	SpO₂, FiO₂, PEEP, respiratory rate, work of breathing, ventilator synchrony, airway security	SpO₂ generally ≥90% or patient-specific target; FiO₂ ≤ 0.6; PEEP ≤10 cmH₂O; RR generally <35–40/min; airway stable	SpO₂ 88–90% with rapid recovery, mild tachypnea, or ventilator asynchrony that resolves with rest or reduced intensity	SpO₂ < 88–90%, sustained desaturation, cyanosis, RR > 35–40/min with distress, unstable airway, severe asynchrony, or FiO₂/PEEP escalation	Common ICU mobilization safety anchor; must be individualized in severe brain injury, chronic respiratory disease, and mechanically ventilated patients	(28, 253, 254)
Metabolic tolerance	Lactate trend, acid–base status, glucose control, temperature, visible fatigue, recovery after rest	No worsening lactic acidosis, severe glycemic instability, uncontrolled fever, or disproportionate fatigue; recovery occurs after rest	Limited metabolic reserve, mild fatigue, borderline glucose/temperature control, or delayed but complete recovery after rest	Worsening acidosis or lactate, severe glycemic instability, uncontrolled fever, disproportionate fatigue, or failure to recover after activity	Indirect but clinically relevant marker of systemic reserve and activity tolerance; especially useful when physiological stress is not captured by vital signs alone	(255)
Neuromuscular and functional capacity	MRC-SS, handgrip dynamometry, ICU Mobility Scale, Perme ICU Mobility Score, CPAx, peripheral muscle ultrasound when available	Stable or improving strength, activity tolerance, posture control, and mobility score at the current level	Severe fatigue, poor postural control, or limited tolerance requires slower progression or return to the previous level.	Loss of postural control, functional decline, inability to recover after rest, or unsafe assistance requirement	Links physiological readiness to measurable functional progression and addresses the need for validated bedside mobility scales.	(46)
Device and line safety	EVD/drain status, airway, vascular access, monitoring cables, staffing, equipment	EVD, drains, airway, vascular access, and monitoring lines secured; staff and equipment adequate	Mobilization is limited by device position, drain management, or staffing constraints, but feasible with modification.	Unsecured airway, unstable EVD/drain, unsafe line configuration, uncontrolled CSF/ICP-related device issue, or inadequate staff support	Operational safety requirement for ICU mobilization feasibility: device safety should be confirmed before every progression step	(11, 28)
Acute ischemic stroke modifier	Neurological deficits, reperfusion status, collateral/perfusion dependence, BP targets, response to head elevation	Stable deficits; no worsening with head elevation; individualized perfusion targets maintained	First 24 h, uncertain reperfusion/collateral status, or perfusion-sensitive symptoms requiring low-intensity and short-duration activity	High-dose or prolonged mobilization within the first 24 h, fluctuating perfusion-dependent deficits, or neurological worsening during activity	Pathology-specific modifier; early mobilization should account for penumbral vulnerability, impaired autoregulation, and collateral-flow dependence	(256)
Intracerebral hemorrhage modifier	Time from onset, BP stability, neurological status, hematoma expansion risk, ICP/mass effect	Usually, after early stabilization, preferably after 24–48 h, when BP and neurological status are stable, and no expansion is evident.	Stable mild/moderate ICH with limited physiological reserve or uncertain expansion risk requiring graded bedside-to-upright progression	First 24 h with active expansion risk, unstable BP, mass effect, raised ICP, or clinical/radiological deterioration.	Addresses hemorrhage-specific feasibility; mobilization should be delayed or restricted during active expansion or intracranial instability.	(257)
Aneurysmal SAH modifier	Aneurysm status, EVD/ICP stability, hydrocephalus, vasospasm/DCI surveillance, CPP	After aneurysm securing, with stable ICP/CPP and no active DCI or hydrocephalus instability	Vasospasm-risk period requiring perfusion-guided, low-intensity, closely monitored progression	Unsecured aneurysm, rebleeding concern, worsening vasospasm/DCI, unstable EVD/ICP, or hydrocephalus instability	Differentiates unsecured from secured aneurysm states and preserves caution during the vasospasm/DCI window.	(258)
Traumatic brain injury modifier	ICP/CPP response, intracranial compliance, sedation/osmotherapy needs, ventilation, response to head elevation	Controlled ICP/CPP, stable ventilation, and tolerated head elevation or low-level activity	Reduced intracranial compliance but stable response to graded positioning and close monitoring	ICP/CPP instability, escalating sedation/osmotherapy, or mobilization-induced intracranial decompensation	Pathology-specific modifier; restrictive intracranial compliance and impaired autoregulation define the mobilization boundary	(4)

### 8.3 Multidisciplinary coordination

Multidisciplinary coordination in neurocritical care reflects the integration of diverse clinical perspectives required to align mobilization strategies with the evolving and patient-specific physiological state (259). Early mobilization does not occur in isolation. Its implementation depends on coordinated input from multiple disciplines, including neurology, critical care medicine, nursing, physiotherapy, and respiratory therapy (260), each contributing distinct and complementary information regarding neurological function, hemodynamic stability, respiratory status, and functional capacity (259, 260). Under optimal conditions, these inputs converge to support coherent and consistent decision-making. In practice, however, variability in the timing of assessments, differences in communication pathways (261), and competing clinical priorities may introduce inconsistencies in how mobilization is initiated, progressed, and adapted (262, 263). As a result, the effectiveness of mobilization strategies is influenced not only by patient physiology but also by the degree of coordination within the clinical team (263). Operationally, the implementing team should be defined according to both medical safety authority and movement-prescription expertise (264). In many neurocritical care settings, the decision to initiate mobilization is made in consultation with the intensivist and neurologist or neurosurgeon, particularly when systemic stability, intracranial dynamics, cerebral perfusion, airway safety, vasopressor use, seizure control, hemorrhage risk, aneurysm status, or postoperative restrictions remain active concerns (16, 264, 265). However, leadership structures vary across healthcare systems, and in some regions, mobilization pathways are primarily led by rehabilitation teams (265). Once medical feasibility is confirmed, the physiotherapist or physical therapist is the most appropriate professional to prescribe, dose, progress, regress, or stop movement because this role integrates functional assessment, activity selection, assistance level, transfer capacity, balance, gait readiness, and tolerance monitoring (266). Nursing staff remains central to bedside implementation through continuous surveillance, line and device safety, positioning, transfer assistance, delirium prevention, and coordination of mobilization with routine ICU care (16, 267, 268). Respiratory therapists, when available, contribute to ventilator readiness, airway security, oxygenation, secretion management, and respiratory response monitoring during activity. These role-specific responsibilities are summarized in Table 8. Multidisciplinary coordination requires integration of neurological, medical, nursing, respiratory, and rehabilitation assessments, with clearly defined responsibilities for mobilization initiation, progression, interruption, and reassessment (11, 77). Neurological assessment may emphasize longer-term recovery trajectories, whereas hemodynamic evaluation often focuses on immediate physiological stability, and rehabilitation planning is directed toward progressive functional improvement (272). Aligning these perspectives requires continuous exchange of information and a shared interpretative framework regarding patient status. When such alignment is achieved, mobilization strategies can be adapted in a manner that reflects both current tolerance and anticipated progression (273). When coordination is fragmented, conflicting signals may delay initiation, restrict progression, or lead to inconsistent application of mobilization practices across care episodes (274). Effective coordination is further constrained by structural factors, including differences in clinical workflow, documentation systems, and communication infrastructure (275). Information may be incomplete, delayed, or interpreted differently across team members, thereby reducing coherence in decision-making processes (276). In addition, standardized protocols may not fully accommodate patient-specific variability, necessitating ongoing adjustment that depends more on team integration than on protocol adherence alone (275, 276). Consequently, the consistency and effectiveness of mobilization implementation are closely linked to the degree to which multidisciplinary inputs are integrated into a unified and adaptive clinical approach.

**Table 8:** Role-based implementation framework for early mobilization in neurocritical care.

Team member	Core responsibility	Role in mobilization progression	References
Intensivist/critical care physician	Confirms systemic and organ-support stability	Determines feasibility when hemodynamics, ventilation, vasopressors, sedation, or systemic instability are concerns	(252)
Neurologist/neurosurgeon	Confirms neurological and intracranial safety	Assesses neurological stability, ICP/CPP status, hemorrhage risk, aneurysm status, vasospasm, seizures, EVD status, and postoperative restrictions	(269)
Physiotherapist/physical therapist	Leads movement prescription	Selects activity level, dose, assistance level, progression, regression, and stopping point after medical feasibility is confirmed	(245)
Nursing staff	Ensures bedside safety and continuity	Coordinates timing, secures lines and drains, monitors tolerance, assists positioning and transfers, and detects early instability.	(240)
Respiratory therapist	Supports airway and ventilatory safety	Evaluates airway security, ventilator synchrony, oxygenation, FiO₂/PEEP tolerance, secretion burden, and respiratory response during activity	(270)
Rehabilitation team leader/mobility coordinator	Coordinates protocol delivery	May lead mobilization pathways in rehabilitation-led systems and standardize documentation, staffing, equipment, and scale-based progression	(271)

### 8.4 Safety thresholds and contraindications

Safety thresholds and contraindications in early mobilization reflect the need to define operational boundaries within which physiological systems can tolerate externally imposed demand without precipitating instability or harm. These thresholds should not be interpreted as fixed numerical values (7). Rather, they represent context-dependent limits shaped by the interaction between hemodynamic status, neurological condition, respiratory function, and metabolic reserve. Under relatively stable conditions, graded activity can be introduced within a range that maintains adequate perfusion, ventilation, and neural responsiveness (277). In neurocritical illness, however, this range is often narrowed. Even modest increases in activity may exceed adaptive capacity when underlying systems are already operating near their limits, making the identification of tolerance boundaries central to safe and effective implementation (77). A key consideration lies in distinguishing between transient physiological responses and patterns that indicate inadequate tolerance (7). Changes in heart rate, arterial pressure, respiratory pattern, or level of consciousness may occur during mobilization without necessarily indicating harm or instability (7, 278). The clinical challenge lies in recognizing patterns that suggest progressive or sustained instability rather than short-lived adaptive responses. This requires interpretation of trends over time rather than reliance on isolated measurements, as well as consideration of how multiple physiological systems respond in combination (279). Contraindications, therefore, extend beyond absolute thresholds and include evolving conditions in which coordinated system responses are insufficient to sustain increasing demand, even when individual parameters remain within nominal ranges (280, 281). Operationalizing safety thresholds is constrained by variability in patient presentation and the limited specificity of available monitoring tools. Standardized criteria may fail to detect early signs of deterioration or may overestimate risk in patients who are capable of tolerating controlled progression (258). In addition, thresholds derived from protocol-based frameworks may not fully reflect the dynamic and non-linear nature of recovery in neurocritical populations. As a result, safety-related decisions are frequently guided by integrated clinical judgment, in which tolerance is inferred from patterns of response across multiple systems rather than from adherence to fixed cutoff values (12, 197, 278). Although mobilization thresholds should remain individualized, the concept of threshold-regulated mobilization requires operational bedside boundaries. These values should not be interpreted as rigid universal cutoffs, but as practical reference ranges that help distinguish feasible mobilization from cautious progression or temporary deferral (282, 283). In neurocritical care, mobilization should generally be considered only when neurological status is stable, intracranial pressure and cerebral perfusion pressure remain within acceptable treatment targets, oxygenation and ventilatory support are stable, and cardiovascular parameters show no sustained decompensation (13, 28, 284). Conversely, mobilization should be deferred or stopped when activity produces sustained neurological worsening, uncontrolled intracranial hypertension, inadequate cerebral perfusion, respiratory failure, escalating vasoactive support, or evidence of active hemorrhagic or ischemic instability (13, 28, 282–284). These operational feasibility, caution, and deferral criteria are summarized in Table 7.

### 8.5 Protocol variability across centers

Protocol variability across centers reflects differences in how early mobilization is conceptualized, structured, and implemented within distinct institutional environments, shaped by available resources, clinical culture, and organizational infrastructure. Although general principles of early mobilization are widely recognized, their translation into clinical practice varies considerably in terms of criteria for initiation, strategies for progression, and thresholds for modification or discontinuation (11, 181). Differences in staffing models, levels of training, and access to rehabilitation services further influence how protocols are applied in practice. As a result, patient populations with similar clinical profiles may experience substantially different mobilization pathways depending on the care environment in which they are treated, introducing variability that extends beyond individual physiological differences (12). A central consideration lies in the balance between standardization and adaptability. Protocol-driven approaches aim to reduce inconsistency and enhance safety by providing structured guidance for initiation and progression. However, strict standardization may not fully accommodate patient-specific variability, particularly in neurocritical populations where tolerance to activity is highly dynamic and context-dependent (285). Conversely, highly individualized approaches may enhance responsiveness to patient needs but introduce variability in clinical decision-making and limit reproducibility across settings. Effective protocols, therefore, function not as rigid algorithms but as flexible frameworks that allow adaptation within clearly defined boundaries (12, 285). Comparisons across centers are further complicated by differences in documentation practices, outcome definitions, and monitoring strategies. Reported protocol adherence may not fully reflect bedside implementation because mobilization definitions, staffing structures, safety criteria, documentation practices, and outcome measures vary across adult ICU and neurocritical-care settings (11, 127). In addition, institutional factors such as workflow constraints, communication patterns, and resource availability influence how protocols are enacted in real time. Consequently, variation in reported mobilization outcomes may reflect differences in protocol design, staffing, documentation, patient selection, monitoring intensity, and organizational context in addition to true differences in intervention effects (11, 12).

## 9 Artificial intelligence and future perspectives

### 9.1 Integration of monitoring and clinical data

The integration of monitoring and clinical data in neurocritical care represents a central challenge in translating fragmented physiological signals into coherent and actionable clinical decision-making, particularly in the context of early mobilization (286). Throughout the course of clinical management, information related to hemodynamic status, cerebral perfusion, respiratory function, and neurological responsiveness is continuously generated across multiple systems and over different temporal scales (287). These data streams are frequently evaluated in isolation, with limited capacity to capture their dynamic interdependence and interaction (287, 288). As a result, clinical decisions regarding mobilization often rely on incomplete or partial representations of the patient’s physiological state, in which underlying instability or latent limits of tolerance may not be fully appreciated or identified (289). Artificial intelligence offers a conceptual and computational framework for integrating these multidimensional inputs into unified and interpretable representations of patient status (290). By combining continuous monitoring signals with structured clinical observations, machine-learning approaches may help identify temporal patterns and cross-system relationships that are not readily detectable through conventional assessment (275, 291, 292). In principle, this may include detecting subtle shifts in physiological trajectories, modeling interactions between variables such as arterial pressure, intracranial dynamics, and respiratory function, and estimating the likelihood of physiological tolerance to incremental levels of activity (147, 293). Rather than functioning as a replacement for clinical judgment, such systems can augment interpretive processes by offering context-aware insights that reflect the evolving behavior of interconnected physiological systems (294). The potential clinical implications of this approach lie in the development of adaptive decision-support tools that align mobilization strategies with patient-specific physiological readiness (295). The integration of multimodal data may facilitate more precise identification of thresholds for initiation, support progression decisions based on real-time patterns of response, and enable earlier detection of instability before overt clinical signs become apparent (295). Within this framework, mobilization may be conceptualized as a continuously modulated process informed by dynamic feedback rather than as a sequence of actions governed by fixed or static protocols (188). Despite these possibilities, implementation remains constrained by several factors, including data heterogeneity, variability in monitoring practices, and challenges related to model interpretability (296). Differences in data quality and acquisition methods across centers, as well as the limited generalizability of models trained in specific contexts, may restrict broader clinical applicability (297). Furthermore, the integration of algorithmic outputs into existing clinical workflows requires careful consideration to prevent overreliance on automated interpretation (296, 297). For these reasons, the role of artificial intelligence is most appropriately viewed as an evolving adjunct to clinical reasoning, the effectiveness of which depends on the quality of integration between data sources, model outputs, and clinician expertise (296, 297).

### 9.2 Decision-support for individualized mobilization

Decision-support for individualized mobilization reflects the need to align intervention strategies with patient-specific physiological capacity rather than applying uniform or protocol-driven approaches across heterogeneous populations (295, 298). In neurocritical care, variability in hemodynamic stability, cerebral perfusion, respiratory function, and neuromuscular responsiveness creates a context in which tolerance to mobilization differs markedly between patients and evolves within the same individual (298). Conventional decision-making approaches rely on discrete clinical observations and predefined thresholds, which may not fully capture the dynamic interactions between these systems (299). As a result, decisions regarding initiation and progression of mobilization are frequently based on incomplete representations of physiological readiness and adaptive capacity (300). Artificial intelligence offers an approach to refine decision-support by integrating multidimensional data into individualized profiles of risk and tolerance. By incorporating continuous monitoring signals alongside clinical variables, predictive models can estimate the likelihood that a given patient will maintain stability during graded activity (299, 300). These models may detect patterns that precede overt clinical deterioration, including subtle changes in hemodynamic variability, alterations in respiratory effort, or shifts in neurological responsiveness (298, 300). Within this context, decision-support systems do not replace clinical judgment, but instead provide probabilistic insights into how an individual patient may respond to incremental changes in activity (299). The clinical value of such approaches lies in their potential to support adaptive and responsive mobilization strategies. Rather than relying on fixed thresholds, mobilization can be dynamically adjusted based on evolving patterns of tolerance, allowing initiation to occur when sufficient stability is present and progression to be modulated according to real-time physiological response (297, 300, 301). This approach may reduce both premature escalation and unnecessary delay, thereby improving alignment between intervention intensity and underlying physiological capacity. Despite these potential advantages, implementation remains limited by challenges related to model validation, data standardization, and integration into routine clinical workflows (297, 302). Predictive accuracy depends on the quality, completeness, and representativeness of the data used for model training, while variability across centers may reduce generalizability. In addition, the interpretability of model outputs is essential for clinical acceptance, particularly in high-risk environments where decision-making must remain transparent and accountable (302, 303). Consequently, decision-support systems should be regarded as complementary tools that assist, rather than direct, clinical reasoning, with their effectiveness determined by how effectively they are integrated into multidisciplinary care processes.

### 9.3 Validation and standardization constraints

Validation and standardization constraints in the application of artificial intelligence to neurocritical care reflect the broader challenge of translating data-driven models into reliable and generalizable clinical tools across heterogeneous healthcare settings (300). Model development is often based on datasets collected within specific institutional environments, where monitoring practices, patient populations, and intervention protocols differ from those encountered in other clinical contexts (304). As a result, model performance observed during development or internal validation may not be maintained when applied to external populations. This limitation is particularly relevant in the context of early mobilization, where physiological responses are shaped by interactions across multiple systems and vary according to injury type, clinical condition, and care pathway (305). A central issue lies in the absence of standardized definitions for both input variables and outcome measures (305). Physiological signals may be recorded using different devices, sampling frequencies, and preprocessing approaches, while clinical endpoints such as tolerance, progression, or functional recovery are defined inconsistently across studies (306, 307). These differences complicate model training, limit comparability, and hinder external validation efforts. Without harmonized data structures and shared criteria for outcome assessment, it becomes difficult to determine whether observed model performance reflects true predictive capability or context-specific patterns embedded within the training dataset (308). Establishing robust validation frameworks requires approaches that explicitly account for temporal dynamics and variability across clinical settings. External validation in diverse populations, prospective evaluation under real-world conditions, and ongoing recalibration of models are necessary to ensure both stability and reliability (309–313). However, these processes are resource-intensive and may be constrained by limitations in data availability, infrastructure, and interoperability between systems. As a result, the translation of artificial intelligence into clinical decision-support for early mobilization remains dependent on the development of standardized data practices and validation strategies capable of accommodating the complexity and variability inherent to neurocritical care populations (300, 308, 314, 315).

## 10 Conclusion

This review advances a system-level interpretation of early mobilization in neurocritical care as a threshold-regulated intervention rather than a uniformly beneficial task-based practice. Across neuromuscular, cerebrovascular, cardiopulmonary, and metabolic–inflammatory domains, current evidence indicates that immobility contributes to disruption of the alignment between neural activation, vascular regulation, oxygen transport, substrate utilization, and functional capacity. Graded reactivation can provide structured physiological input to these disrupted systems, engaging processes related to neurovascular coupling, systemic hemodynamic adaptation, skeletal muscle metabolism, respiratory function, and activity-dependent recovery. However, the magnitude, direction, and clinical relevance of these responses depend on injury type, timing, mobilization dose, cerebral autoregulatory integrity, systemic physiological reserve, and the patient’s tolerance during and after activity. A central conclusion of this review is that demonstrated clinical effects and mechanistically plausible pathways should not be interpreted with the same level of certainty. Clinical studies support the feasibility and potential safety of selected early mobilization strategies in appropriately screened critically ill or neurocritical patients, but the evidence remains heterogeneous with respect to population, timing, intervention intensity, monitoring strategy, and outcome definition. In contrast, many pathways discussed in this review, including neurovascular recoupling, mitochondrial bioenergetic adaptation, inflammatory modulation, synaptic responsiveness, and network-level cognitive recovery, remain biologically plausible or mechanistically inferred unless directly evaluated in acute neurocritical populations. Maintaining this distinction is essential for accurate interpretation and for avoiding overextension of findings derived from experimental, non-neurocritical, chronic neurological, or general ICU settings. Translation into consistent clinical practice remains constrained by several unresolved challenges. The operational boundaries within which mobilization is both safe and potentially beneficial are not yet clearly defined for different neurocritical pathologies. Uncertainty persists regarding autoregulatory windows, intracranial pressure and cerebral perfusion pressure tolerance, regional perfusion vulnerability, respiratory reserve, metabolic response, and recovery after activity. Commonly used bedside indicators often provide fragmented or delayed representations of integrated system behavior, limiting the ability to identify transient instability or compensatory adaptation in real time. In addition, variability in mobilization protocols, biomarker selection, adverse-event definitions, and functional outcome measures continues to limit reproducibility and complicate causal interpretation. Future research should prioritize three areas. First, prospective studies should evaluate threshold-informed mobilization protocols that incorporate neurological status, intracranial dynamics, cerebral perfusion, systemic hemodynamics, respiratory reserve, metabolic tolerance, functional capacity, and device safety into explicit initiation, progression, modification, and stopping criteria. Second, standardized outcome definitions and adverse-event reporting frameworks are needed to improve comparability across neurocritical care settings. Third, integrated monitoring approaches should be developed and validated to capture cross-system interactions between cerebral perfusion, systemic physiology, respiratory function, metabolic demand, and functional response during mobilization.

Overall, early mobilization in neurocritical care is best understood as an individualized, physiology-guided clinical strategy. Its value depends not simply on initiating activity early, but on matching the timing, intensity, and progression of mobilization to patient-specific tolerance thresholds. This threshold-regulated framework provides a clinically useful way to integrate mechanistic rationale, bedside monitoring, safety constraints, and functional goals under conditions of neurological and physiological instability.
