# Supplementary material for: Early mobilization in neurocritical care as a threshold-regulated intervention: a narrative review of mechanistic integration and implications for neurological recovery and physiological stability
Source: Front Neurol. 2026 Sep 29;17:1837085. doi: 10.3389/fneur.2026.1837085 (PMC13659806; doi:10.3389/fneur.2026.1837085)
Supplement: Supplementary file 1 [file Table_1.DOCX]

**Table S1. Contraction-driven metabolic signaling pathways during early mobilization**

| **Mechanistic Module** | **Signal Origin** | **Signal Class** | **Core Molecular Mediators** | **Target Interface** | **Primary Signaling Pathway** | **Functional Outcome (Context-Dependent)** | **Dominant Activation Niche** | **Biological Implication** | **Challenges** | **References** |
| --- | --- | --- | --- | --- | --- | --- | --- | --- | --- | --- |
| **Contraction-induced calcium signaling** | Sarcoplasmic reticulum release | Ionic flux | Ca²⁺, calmodulin, CaMKII | Mitochondria, nucleus | Ca²⁺/CaMK signaling | Partial activation of mitochondrial enzymes | Early low-intensity activation | Supports oxidative readiness | Indirect measurement in vivo | (115,116) |
| **Energy-sensing activation** | ATP turnover during contraction | Metabolic stress signal | AMPK, ADP/ATP ratio | Cytosol, mitochondria | AMPK pathway | Increased glucose uptake, metabolic adjustment | Low–moderate intensity activity | Enhances metabolic flexibility | Context-dependent activation threshold | (117,118) |
| **Mitochondrial biogenesis signaling** | Repeated contractile activity | Transcriptional activation | PGC-1α, NRF1 | Nuclear–mitochondrial axis | PGC-1α pathway | Partial mitochondrial adaptation | Sustained activation phases | Supports energy production capacity | Delayed temporal response | (119) |
| **Glucose transport regulation** | Muscle contraction | Transport signaling | GLUT4, insulin-independent pathways | Cell membrane | AMPK–GLUT4 translocation | Improved glucose uptake efficiency | Acute mobilization phase | Reduces insulin resistance tendency | Difficult to isolate from systemic effects | (120,121) |
| **Proteostasis modulation** | Mechanical load | Anabolic–catabolic balance | mTOR, ubiquitin–proteasome components | Cytoskeleton, ribosomes | mTOR / proteolysis pathways | Partial restoration of protein balance | Variable with loading intensity | Preserves muscle structure | Overlap with inflammation effects | (122,123) |
